# Advancements in Clinical Applications of Transcranial Magnetic Stimulation Using Different Coil Configurations: A Review

**DOI:** 10.3390/bioengineering13101214

**Published:** 2026-10-19

**Authors:** Wentao Zhang, Mai Lu

**Affiliations:** Key Laboratory of Opto-Electronic Technology and Intelligent Control of Ministry of Education, Lanzhou Jiaotong University, Lanzhou 730070, China; by_zhwt@126.com

**Keywords:** TMS, coil configuration, neuromodulation, neurological disorder, psychiatric disorder

## Abstract

The effects of transcranial magnetic stimulation (TMS) are jointly determined by coil geometry, intracranial electric-field distribution, stimulation target, and pulse parameters, with coil design directly governing stimulation depth, spatial focality, and brain tissue coverage. This review compares circular, figure-of-eight, double-cone, and H-coils in terms of geometry, electric-field distribution, effective depth, focality, and current clinical use. Figure-of-eight coils provide high superficial focality and are suited to anatomically defined cortical targets such as the dorsolateral prefrontal cortex, primary motor cortex, and supplementary motor area. Double-cone and H-coils extend effective electric-field coverage to medial and relatively deep networks but reduce spatial selectivity, illustrating the depth–focality trade-off. The maturity of clinical evidence varies across disorders. Conventional repetitive TMS, intermittent theta-burst stimulation, and H1-coil deep TMS have comparatively established evidence in depression, whereas stroke and migraine remain heterogeneous in terms of target selection, dose, and patient selection. Key challenges are to match individualized intracranial electric-field dose, disease-network targets, pulse sequences, and brain state. This work offers valuable guidance for researchers in related fields and professionals engaged in the clinical management of neuropsychiatric disorders.

## 1. Introduction

Transcranial magnetic stimulation (TMS) is a noninvasive neuromodulation technique in which a rapidly changing magnetic field passes through the scalp and skull and induces an electric field in brain tissue, thereby modulating cortical excitability and neural-network activity [1]. Since Barker et al. first achieved noninvasive magnetic stimulation of the human motor cortex in 1985, TMS has evolved from an experimental technique for motor-cortex testing and neurophysiological research into a neuromodulation platform with value for both brain-function research and clinical intervention [1]. With the development of stimulation paradigms such as repetitive TMS (rTMS) and theta-burst stimulation (TBS), together with continuing improvements in stimulators, coil configurations, and target-localization methods, TMS has been widely applied in neuroscience research and in interventions for multiple neurological and psychiatric disorders [2,3]. Its development therefore encompasses not only neurophysiology and clinical medicine but also stimulation hardware, electromagnetic-field control, and dose optimization, making TMS a representative interdisciplinary field in biomedical engineering.

The spatial and biological effects of TMS are not determined solely by stimulation frequency or stimulator output. They depend strongly on coil geometry, the magnitude and direction of the intracranial induced electric field, coil-to-cortex distance, effective depth, and spatial focality [4,5]. Different coil configurations determine how magnetic fields superimpose in space and consequently affect the volume of brain tissue that reaches an effective stimulation threshold. TMS coil design therefore faces a fundamental engineering constraint: the depth–focality trade-off [4]. This relationship is not determined by the coil alone. Individual anatomical factors, including gyral and sulcal morphology, tissue conductivity, head curvature, coil orientation, and scalp-to-cortex distance, also alter electric-field magnitude, direction, and peak location at the target [5]. Simply increasing stimulator output cannot increase stimulation depth indefinitely while maintaining the safety of superficial tissues. TMS should instead be understood as an electromagnetic neuromodulation system jointly determined by coil geometry, the intracranial electric field, and stimulation dose [4,6].

Although TMS research has developed rapidly, the literature often addresses the technology from separate perspectives. Clinical reviews and evidence-based guidelines primarily focus on therapeutic efficacy, stimulation frequency, intensity, and safety across neuropsychiatric disorders [3,7]; biophysical and computational studies emphasize intracranial electric-field modeling, tissue conductivity, and field-distribution patterns [6]; and coil-engineering studies compare geometries in terms of depth, focality, and energy efficiency [4]. These lines of research have advanced clinical application and engineering design, yet the continuous relationship among coil geometry, intracranial field distribution, target accessibility, stimulation paradigm, and clinical indication still requires integration within a unified framework. Highly focal coils may be advantageous for superficial, anatomically well-defined motor or prefrontal targets. For disease networks involving several cortical nodes and deeper functional connections, however, maximizing local field focality may not achieve adequate network modulation. Conversely, increasing depth and coverage may recruit a broader disease-related network at the cost of anatomical selectivity [4,8]. Re-examining TMS coils through the correspondence between engineering performance and clinical need is therefore important for understanding their technical advantages, indications, and limitations.

This review systematically compares representative circular, figure-of-eight, double-cone, and H-coils used in conventional and deep TMS (dTMS), with an emphasis on how coil geometry affects intracranial electric-field magnitude, spatial distribution, effective depth, and focality. Figure-of-eight coils superimpose the fields generated by adjacent windings and thereby provide high superficial spatial focality, which makes them suitable for relatively well-defined cortical targets such as the primary motor cortex and dorsolateral prefrontal cortex [9]. Double-cone coils extend field coverage to medial and relatively deep brain regions through their angled two-wing structure [10]. H-coils use three-dimensional distributed windings to expand depth and coverage but have lower spatial focality [8,11]. Building on these physical comparisons, we relate coil-specific field distributions to stimulation targets, pulse sequences, and disease-related neural networks in representative neurological and psychiatric disorders. By integrating coil design, electric-field dose, target selection, stimulation parameters, and clinical indication in an engineering–clinical framework, this review aims to clarify the engineering basis and practical boundaries of current TMS coil designs and to inform future precision-neuromodulation strategies based on individualized field dosing, disease-network targeting, and image-guided neuronavigation.

We searched PubMed/MEDLINE, Embase, Web of Science, Scopus, and the Cochrane Library from database inception through 8 August 2026. Search concepts combined interventions such as “transcranial magnetic stimulation,” “repetitive transcranial magnetic stimulation,” “theta-burst stimulation,” “deep TMS,” and “H-coil” with disease and outcome terms including “depression,” “treatment-resistant depression,” “neuroplasticity,” “efficacy,” and “safety”; controlled vocabulary was adapted for each database. Eligible sources included randomized controlled trials, observational studies, systematic reviews, meta-analyses, guidelines, expert consensus statements, and studies of stimulation mechanisms or coil technology. We excluded duplicates, sources unrelated to the review question, reports for which the full text was unavailable, and promotional webpages without verifiable research data. Study selection considered the population, TMS intervention, comparator, outcomes, and study quality.

## 2. Historical Development of Transcranial Magnetic Stimulation

The modern development of TMS is generally traced to Barker et al.’s successful noninvasive stimulation of the human motor cortex in 1985. Early TMS systems mainly used circular coils, which induced cortical electric fields over a broad area but offered limited spatial focality [1]. In 1988, Ueno et al. introduced the figure-of-eight coil. The induced fields generated by its two adjacent windings superimpose at their junction, producing a more concentrated peak in the superficial cortex, markedly improving spatial resolution, and eventually establishing this geometry as the principal coil configuration for functional mapping and conventional therapeutic TMS [9]. This stage marked a shift from asking whether brain tissue could be activated to controlling the stimulation site, affected volume, and field distribution with greater precision.

After the 1990s, TMS expanded from single-pulse neurophysiological testing to modulation of cortical excitability and clinical treatment. Paired-pulse TMS and rTMS made stimulation frequency, intensity, pulse number, and temporal pattern key determinants of neuromodulatory effects [12,13,14]. In 2005, Huang et al. introduced TBS, broadening the temporal design space and creating a technical route toward shorter treatment sessions [2]. In parallel, coil design progressed from superficial focal stimulation toward modulation of deeper and more distributed neural networks. Double-cone coils improved access to medial and relatively deep cortical regions through angled wings [10], whereas H-coils used three-dimensional distributed windings to increase effective depth and coverage, advancing dTMS [8,11]. These developments also exemplified the persistent depth–focality trade-off in TMS coil design.

**Figure 1:**
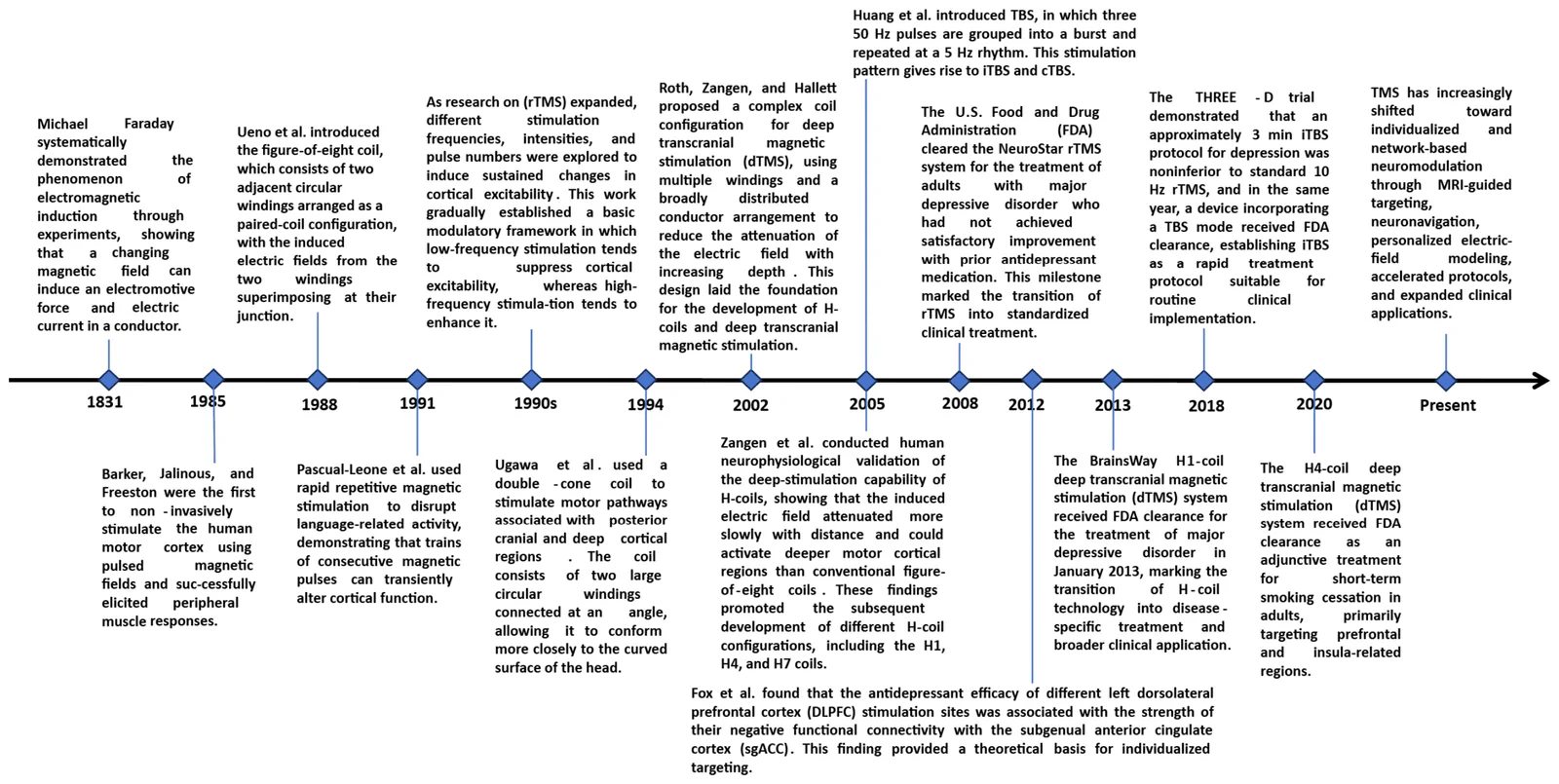
Developmental timeline of transcranial magnetic stimulation (Compiled and illustrated based on refs. [1,2,9,11,13,15,16,17,18,19,20]).

As stimulators, treatment parameters, and clinical-trial methods matured, TMS entered standardized clinical practice. In 2008, the U.S. Food and Drug Administration cleared a specific TMS device for adults with treatment-resistant major depressive disorder, after which devices and protocols expanded to clinical contexts including migraine, obsessive–compulsive disorder, and smoking cessation [15]. More recently, structural and functional magnetic resonance imaging, neuronavigation, individualized electric-field modeling, and accelerated protocols have driven a transition from empirical stimulation based on scalp landmarks toward individualized, network-informed, and quantitatively dosed neuromodulation. Overall, the evolution of TMS can be summarized as a progression from broad superficial stimulation to focal precision stimulation and then to deep-network modulation and individualized treatment. The major technical milestones and translational steps are shown in Figure 1.

## 3. Physical Principles of Transcranial Magnetic Stimulation

The physical basis of TMS is Faraday’s law of electromagnetic induction. When a rapidly changing current pulse flows through the stimulation coil, it produces a transient magnetic field around the coil. Because the magnetic permeability of the scalp and skull is close to that of air, the magnetic field traverses the skull with relatively little attenuation and induces an electric field in brain tissue [21]. The induced electromotive force is described by Faraday’s law, as shown in Equation (1):

(1) $$E=-\frac{d\Phi_{B}}{\mathrm{dt}},$$

**Figure 2:**
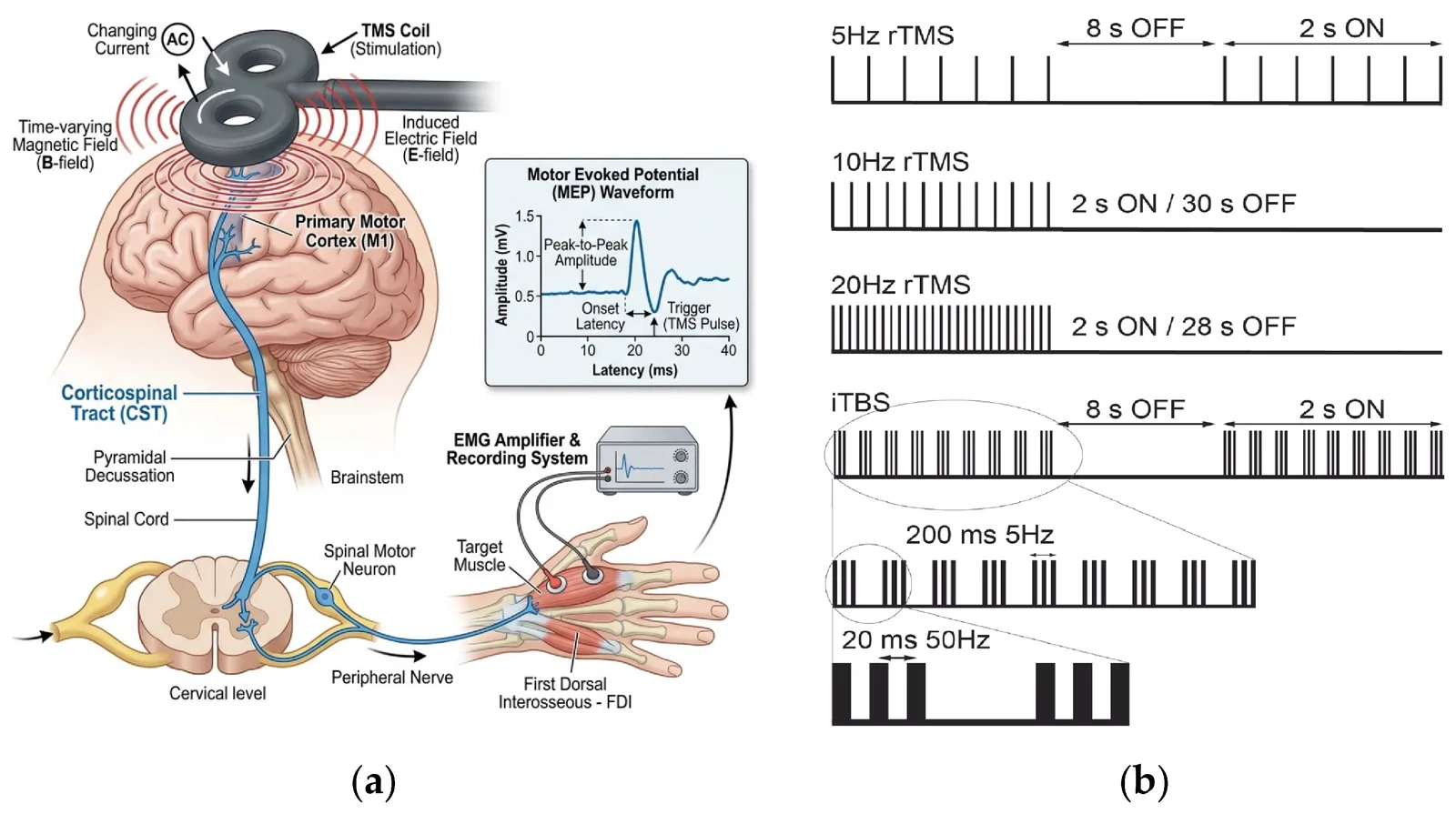
(**a**) Transcranial magnetic stimulation of the motor cortex and spinal pathways, with motor-evoked potentials (MEPs) recorded to quantify stimulation effects. (**b**) Pulse frequencies and intervals commonly used in rTMS protocols. (Adapted with permission from Ref. [23]. Copyright 2025 Benavides, Shine, Stefanovic, Chen and Jo.)

In the volume conductor of the brain, the induced electric field drives directional ionic movement and creates local currents. When the local field is sufficiently strong, it can alter neuronal membrane potential and evoke action potentials, thereby noninvasively modulating cortical excitability and neural-network activity [6]. As shown in Figure 2a, TMS can stimulate the motor cortex while motor-evoked potentials (MEPs) are recorded to assess corticospinal excitability and the effects of stimulation. The practical action of TMS therefore depends not only on stimulator output but also on coil geometry, the rate of current change, the coil-to-cortex distance, and local tissue electrical properties.

The intracranial induced electric field can be divided conceptually into primary and secondary components. The primary field is generated directly by the time-varying magnetic field and is governed by coil geometry and current characteristics. The secondary field arises mainly from charge accumulation at interfaces between tissues of different conductivity. Because gray matter, white matter, cerebrospinal fluid, and the skull have different conductivities, field distribution in a realistic head is strongly shaped by tissue boundaries, gyral and sulcal morphology, and head geometry [6]. The secondary field has been reported to reach approximately 20–35% of the primary field [22] and therefore should not be neglected in individualized field modeling or target-dose assessment.

In addition to spatial field distribution, the temporal pulse pattern is a major determinant of neuromodulation. rTMS uses repeated pulse trains to induce persistent modulation: high-frequency rTMS generally tends to facilitate cortical excitability, whereas low-frequency rTMS more often exerts inhibitory effects. TBS combines high-frequency pulse bursts with a lower-frequency rhythm to induce plasticity within a shorter period [2]. Intermittent TBS (iTBS) is usually facilitatory, whereas continuous TBS (cTBS) is more often inhibitory, although the actual response remains dependent on stimulation intensity, baseline brain state, and interindividual variability. Common stimulation modes, typical frequencies, regulatory tendencies, and clinical applications are summarized in Table 1; representative temporal patterns of rTMS and TBS are shown in Figure 2b.

Stimulation frequency and temporal sequence should therefore not be treated as isolated variables but rather interpreted together with coil configuration, target location, and electric-field dose. From a translational perspective, coil geometry determines the spatial distribution of the electric field, stimulation parameters determine the temporal dose, and individual brain structure and functional state determine the resulting neural response. This framework provides the physical basis for the subsequent comparison of circular, figure-of-eight, double-cone, and H-coils in terms of depth, focality, and suitability for clinical targets.

## 4. Clinical Applications of Conventional Transcranial Magnetic Stimulation

The geometry of a TMS coil determines the spatial distribution of the intracranial induced electric field and consequently affects stimulation depth, focality, and effective coverage. Using finite-element modeling to compare 50 commercial and experimental coils, Deng et al. identified a general depth–focality trade-off: increasing depth usually enlarges the tissue volume exposed to an effective field, whereas increasing focality is generally accompanied by faster field decay with depth [4]. TMS coil development has therefore not been a simple pursuit of deeper or stronger stimulation but rather an effort to balance depth, spatial selectivity, and clinical target requirements. Under this design logic, conventional coils evolved from broad-coverage circular coils toward the more focal figure-of-eight geometry.

### 4.1. Conventional Two-Dimensional Coil Design

#### 4.1.1. Circular Coil

**Figure 3:**
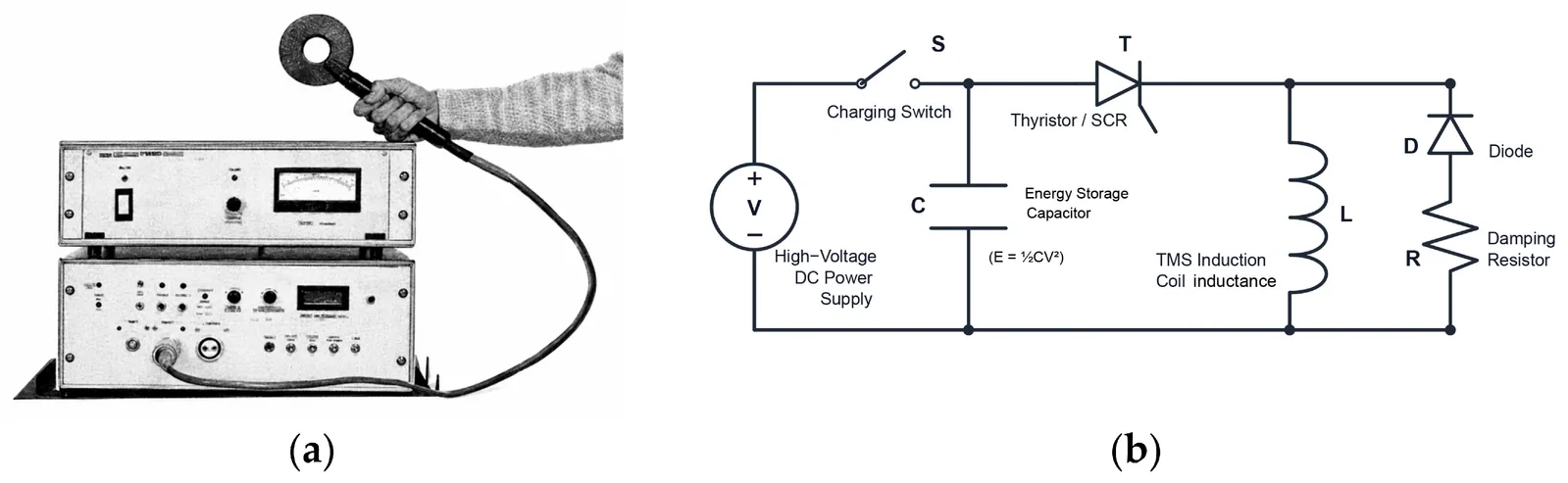
(**a**) Representative early transcranial magnetic stimulation device. (Reprinted with permission from ref. [1]. Copyright 1985 Elsevier.) (**b**) Simplified circuit of a single-pulse stimulator.

Anthony Barker and colleagues developed the first TMS system used in human studies in 1985 [1]. As shown in Figure 3a, the system consisted primarily of a circular copper-wire stimulation coil and a high-voltage capacitor discharge unit. During stimulation, the energy-storage capacitor delivered a brief current pulse with a peak of approximately 4000 A and a duration of about 110 μs, generating a rapidly changing magnetic field and an induced electric field in the brain [1]. At the circuit level, early stimulators can be approximated as resistor–inductor–capacitor discharge circuits (Figure 3b). The rapid change in coil current is essential for generating a strong induced field, and coil inductance, resistance, and stimulator waveform jointly affect energy efficiency, pulse shape, and thermal load [24].

**Figure 4:**
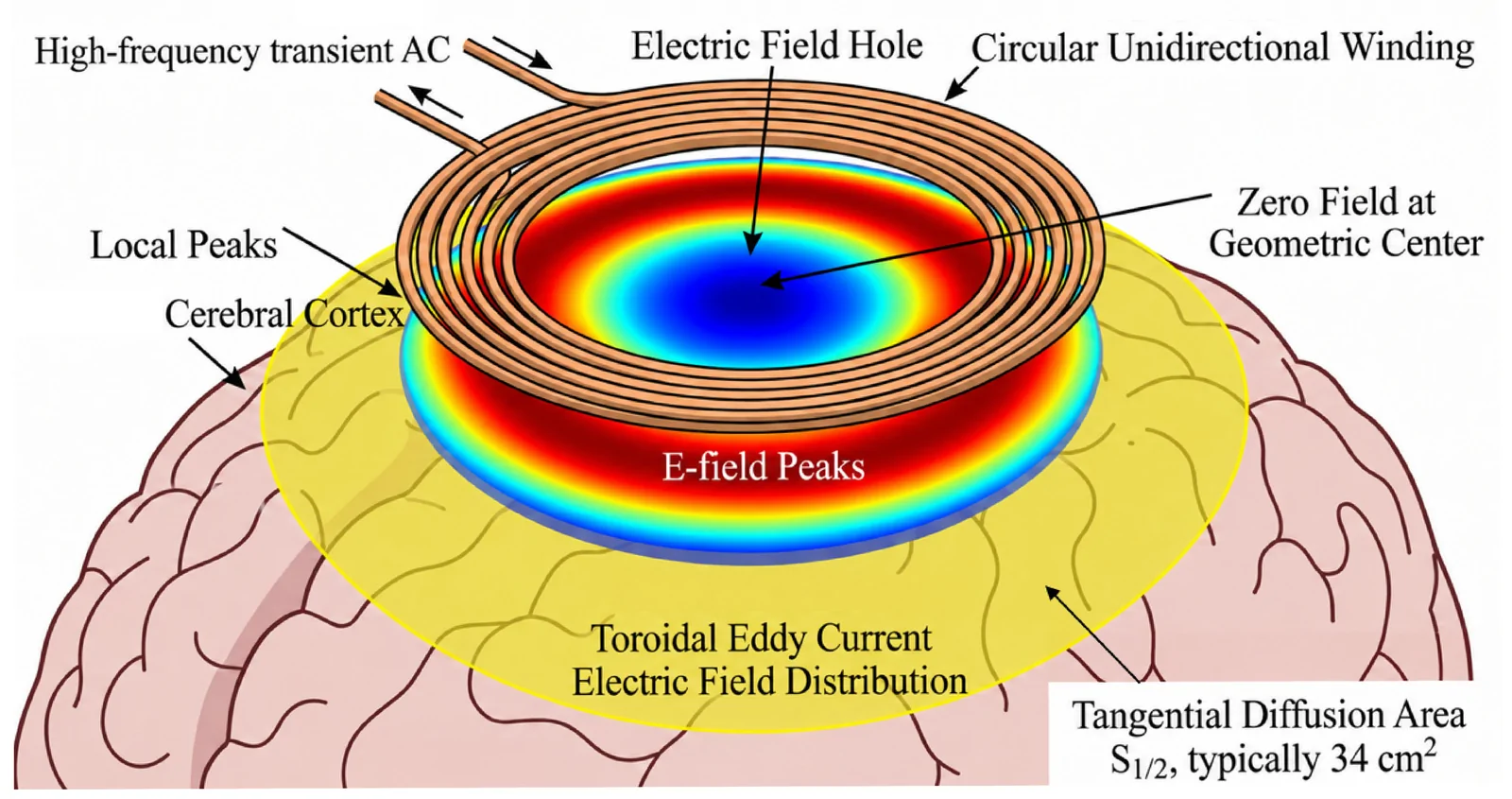
Schematic illustration of the working effect of a circular coil.

A circular coil generally comprises a single axisymmetric circular winding. A transient current produces an annular induced electric field in the underlying brain tissue around the coil axis. Field components of different directions generated by the axisymmetric geometry largely cancel near the geometric center, creating a relatively low-field region directly beneath the center, whereas stronger fields occur around the projected winding. The region of maximal stimulation is therefore annular rather than centered on the coil, as illustrated in Figure 4. Circular coils have a broad tangential spread; modeling studies have reported a half-maximum spread area of approximately 34 cm^2^ [4].

The broad field coverage of circular coils allows simultaneous influence over a relatively large superficial cortical area but limits spatial localization. Depending on coil size and modeling conditions, the half-value depth is approximately 1.0–3.5 cm, yielding an electromagnetic profile of broad coverage and low focality [4,25]. As TMS shifted from neurophysiological testing toward targeted neuromodulation, circular coils were gradually replaced by more focal figure-of-eight coils for localized cortical targets such as the DLPFC and M1. Their broad coverage and tolerance to positioning error nevertheless remain useful for assessing central motor conduction time and peripheral nerve or nerve-root conduction [26]. Circular coils thus provided a foundation for early TMS and a reference for later designs that altered winding number, direction, and spatial arrangement to optimize induced-field distribution.

#### 4.1.2. Figure-of-Eight Coil

**Figure 5:**
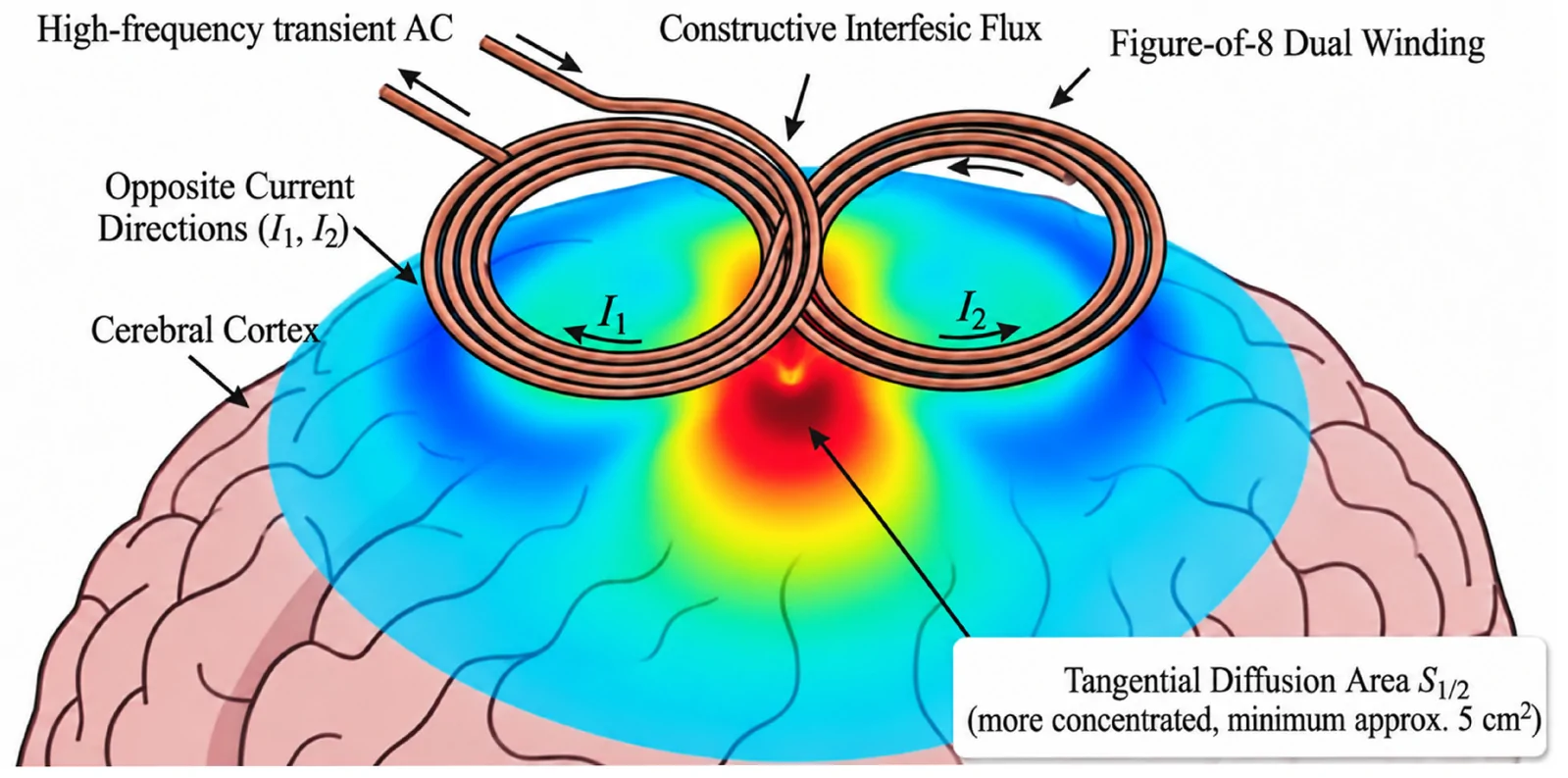
Schematic illustration of the working effect of a figure-of-eight coil.

To improve the limited spatial focality of circular coils, Ueno et al. introduced the figure-of-eight, or butterfly, coil in 1988 [9]. It consists of two adjacent circular windings carrying current in opposite directions. The induced fields from the two windings superimpose at the central junction, creating a localized high-intensity field beneath the intersection, whereas some field components progressively cancel away from the junction. This geometry produces a relatively concentrated superficial stimulation hotspot and substantially improves spatial focality [9,19], as shown in Figure 5.

In 1990, Ueno et al. further applied the figure-of-eight coil to functional mapping of the human primary motor cortex and achieved spatial resolution on the order of 5 mm [27]. Compared with a circular coil, the figure-of-eight coil confines high-intensity induced fields to a smaller superficial cortical area and has therefore become one of the most widely used configurations for brain mapping and conventional therapeutic TMS [4]. In an ideal homogeneous model, the maximum field is usually located beneath the junction of the two windings. In a realistic head, however, gyral and sulcal morphology, tissue conductivity, head curvature, and coil position may shift the field maximum away from the geometric center [5].

In addition to geometry, coil orientation and coil-to-cortex distance strongly influence the practical effects of a figure-of-eight coil. Rotating the coil changes the direction of the central induced current and intracranial field, thereby altering which neuronal populations are preferentially recruited. Different orientations can consequently produce different neurophysiological effects even when the stimulation location is unchanged. The induced field also decays rapidly as the coil-to-cortex distance increases; scalp thickness, skull morphology, the cerebrospinal-fluid layer, and local sulcal anatomy all modify the dose received by the target [5]. Clinical use should therefore maintain stable contact between the coil and scalp and optimize position and orientation with respect to individual head shape and target anatomy.

The principal advantage of the figure-of-eight coil is high superficial focality, but its effective depth is comparatively limited. This feature exemplifies the general depth–focality trade-off: reducing winding size can further improve surface focality but accelerates field decay with depth [4]. Figure-of-eight coils are therefore particularly suitable for anatomically well-defined superficial targets such as the DLPFC, M1, and supplementary motor area (SMA). Deeper or more distributed functional networks therefore require modified or alternative coil configurations. For example, combining a conventional figure-of-eight coil with a large Halo coil can increase field penetration while preserving a relatively concentrated superficial field beneath the figure-of-eight component [28]. Double-cone and H-coils represent other approaches for extending effective field coverage.

### 4.2. Clinical Efficacy of Conventional Transcranial Magnetic Stimulation

#### 4.2.1. Major Depressive Disorder

Major depressive disorder (MDD) is among the clinical indications with the strongest evidence base for TMS. Conventional TMS most often uses a figure-of-eight coil to stimulate the dorsolateral prefrontal cortex (DLPFC), and its high superficial focality allows relatively consistent coverage of this cortical target. Current strategies include high-frequency rTMS over the left DLPFC, low-frequency rTMS over the right DLPFC, sequential bilateral stimulation, and intermittent theta-burst stimulation (iTBS) over the left DLPFC. These protocols exert antidepressant effects by modulating prefrontal excitability and associated emotion-related networks [3,29].

The most representative conventional protocol is 10 Hz rTMS over the left DLPFC. In a large multicenter randomized controlled trial, O’Reardon et al. used an approximately 70 mm figure-of-eight coil to deliver 10 Hz stimulation at 120% of the resting motor threshold (RMT), approximately 3000 pulses per session, for 4–6 weeks; active stimulation was superior to sham [29]. Subsequent studies strengthened the evidence base, and high-frequency left DLPFC stimulation has become an important noninvasive option for patients with insufficient improvement or intolerance to pharmacotherapy [3,29,30]. By comparison, low-frequency right DLPFC rTMS is commonly delivered at 1 Hz, 110–120% RMT, and approximately 600–1600 pulses per session. Some trials and meta-analyses report antidepressant efficacy comparable to left-sided high-frequency protocols; safety guidelines also indicate a relatively low seizure risk [31], which makes it an alternative for selected patients [32,33,34]. Sequential bilateral protocols generally combine 1 Hz right-sided stimulation with 10–20 Hz left-sided stimulation and may outperform sham stimulation [35].To improve treatment efficiency, iTBS has become an important alternative to conventional high-frequency rTMS. Standard iTBS consists of 50 Hz triplet bursts repeated at 5 Hz, commonly delivered at 120% RMT for approximately 600 pulses over about 3 min. The multicenter noninferiority THREE-D randomized trial in 2018 showed that iTBS was noninferior to standard 10 Hz rTMS for antidepressant efficacy while reducing each session from approximately 37.5 min to about 3 min [20]. iTBS is therefore a practical protocol for patients and services seeking to balance efficacy, treatment time, and resource utilization.

Accelerated and individualized stimulation has recently shifted MDD treatment from fixed anatomical targets toward functional-network targeting. Accelerated iTBS approaches such as Stanford Accelerated Intelligent Neuromodulation Therapy (SAINT), also termed Stanford Neuromodulation Therapy (SNT), use functional-connectivity information to select an individualized left DLPFC target and deliver multiple high-dose sessions over a short period; early studies reported rapid and high response and remission rates [36]. These protocols, however, differ substantially from conventional rTMS in terms of dose, sessions per day, imaging and neuronavigation requirements, and patient-selection criteria, and some early studies had limited sample sizes. Their effect magnitude and generalizability therefore require independent validation in larger samples [36,37]. Overall, 10 Hz left DLPFC rTMS and standard iTBS have the most mature evidence among conventional approaches. Right-sided low-frequency and bilateral stimulation are alternatives, whereas accelerated, connectivity-guided individualized TMS represents an important emerging direction. Representative parameters and clinical evidence are summarized in Table 2.

#### 4.2.2. Post-Stroke Motor Recovery and Interhemispheric Inhibition Networks

Post-stroke motor recovery involves network reorganization across both cerebral hemispheres, cortico-subcortical circuits, and the corticospinal tract. The interhemispheric-imbalance model is an important theoretical framework for interpreting abnormal post-stroke motor networks and rTMS mechanisms. Under physiological conditions, the two primary motor cortices (M1) maintain dynamic transcallosal inhibition. After stroke, excitability of ipsilesional M1 and corticospinal output may decline, reducing inhibition of contralesional M1; the contralesional hemisphere may consequently become relatively hyperexcitable and exert excessive inhibition on ipsilesional M1 [38,39]. The principal aim of TMS in motor rehabilitation is therefore not simply to alter local cortical excitability but also to regulate the excitation–inhibition balance between bilateral motor networks.

Two main strategies have emerged from this framework. The first uses low-frequency rTMS or cTBS over contralesional M1 to reduce its excitability and abnormal transcallosal inhibition of the affected hemisphere. The second uses high-frequency rTMS or iTBS over ipsilesional M1 to enhance recruitment of the affected cortex and residual corticospinal system [39,40,41,42]. Common contralesional low-frequency protocols use 1 Hz, approximately 80–100% RMT, and 900–1800 pulses per session. Ipsilesional high-frequency protocols commonly use 5–20 Hz, approximately 80–100% RMT, and about 1000–1500 pulses per session. TBS usually consists of 50 Hz triplet bursts repeated at 5 Hz; cTBS and iTBS commonly use 600 pulses to inhibit contralesional M1 or facilitate ipsilesional M1, respectively [2,43,44,45].

The interhemispheric-imbalance model does not apply to every patient with stroke. When lesions are extensive, the corticospinal tract is severely damaged, or structural reserve in the affected hemisphere is limited, the contralesional hemisphere may provide compensatory support; excessive inhibition of contralesional M1 may then be detrimental. In a randomized sham-controlled study, Kim et al. (2020) found that the added benefit of contralesional low-frequency rTMS was inconsistent in the overall sample, whereas patients without substantial cortical involvement and with predominantly subcortical or brainstem lesions may have experienced greater motor improvement [46]. Qian et al. (2024) further reported that patients with detectable MEPs from the affected hemisphere may respond better to low-frequency rTMS than MEP-negative patients [47]. MEP status, corticospinal-tract integrity, lesion location, and stroke stage may therefore be more useful for stratification than a fixed contralesional-inhibition/ipsilesional-facilitation rule.

Clinical studies suggest that low- and high-frequency rTMS, cTBS, and iTBS may improve Fugl-Meyer scores, Action Research Arm Test (ARAT) performance, and selected fine-motor outcomes. Chang et al. (2010) reported that high-frequency stimulation of ipsilesional M1 promoted motor recovery after subacute stroke [48]. Studies by Kuzu et al. (2021) and Vink et al. (2023) suggested that contralesional cTBS combined with rehabilitation may improve upper-limb function, although the evidence remains limited by sample size and patient heterogeneity [43,44]. Chen et al. (2019) found that ipsilesional iTBS improved upper-limb motor function in chronic stroke [45]. Across studies, however, stroke stage, lesion characteristics, stimulation parameters, rehabilitation content, and outcome measures vary considerably, and rigorously controlled trials have yielded inconsistent results. TBS is shorter than conventional rTMS, but its evidence base remains smaller, and its advantages cannot be generalized to all patients. TMS is therefore better positioned as an adjunct to conventional rehabilitation than as a stand-alone replacement. Future protocols should individualize target and stimulation direction using MEP status, corticospinal-tract integrity, and functional connectivity. Representative studies, parameters, outcomes, and limitations are shown in Table 3.

#### 4.2.3. Migraine

The pathophysiology of migraine involves cortical spreading depression (CSD), the trigeminovascular system, and abnormalities in brainstem–thalamic and cortical pain-modulation networks [50,51]. TMS has followed two broad therapeutic paths. Occipital single-pulse TMS may be used during acute attacks to modulate neural activity associated with CSD and thalamocortical transmission, whereas preventive approaches most often use a figure-of-eight coil over the left M1 or DLPFC. M1 stimulation engages sensorimotor and descending pain-control systems, whereas DLPFC stimulation is thought to modulate pain attention, emotional processing, and descending inhibition through prefrontal–limbic and brainstem networks. Preventive studies have mainly tested high-frequency rTMS at 5–20 Hz, commonly 10 Hz over left M1; iTBS is an exploratory time-efficient alternative.

Clinical evidence suggests that high-frequency rTMS may reduce migraine burden, although results vary across targets and protocols. Studies by Misra et al. (2013) and Shehata et al. (2016) showed that 10 Hz stimulation of left M1 reduced attack frequency and improved selected pain-related outcomes [52,53]. Brighina et al. (2004) reported preventive effects from high-frequency left DLPFC stimulation [54], whereas the randomized trial by Conforto et al. (2014) did not demonstrate a consistent advantage of active stimulation [55]. Sahu et al. (2019) further suggested that left DLPFC iTBS may improve attack frequency, duration, and pain severity, but the evidence is derived mainly from small samples [56]. Systematic reviews and meta-analyses by Moisset et al. (2020) and Zhong et al. (2022) concluded that rTMS may reduce attack frequency and headache days, while the effects on pain intensity, the relative merits of M1 and DLPFC, and the optimal dose remain unsettled [57,58].

The current challenge in migraine is therefore not simply to identify a therapeutic signal but also to determine which patient subtypes, targets, and standardized parameters are most appropriate. Preventive evidence is concentrated on high-frequency stimulation of left M1 or DLPFC with a figure-of-eight coil; 10 Hz M1 protocols have comparatively more clinical support. High-frequency DLPFC stimulation and iTBS may also be valuable, but consistency is limited. TMS is presently best regarded as an adjunctive or investigational neuromodulation option for patients with insufficient benefit from conventional preventive therapy, and standardization remains less mature than in MDD. Representative studies, parameters, outcomes, and limitations are presented in Table 4.

#### 4.2.4. Parkinson’s Disease

Motor symptoms of Parkinson’s disease (PD) are closely associated with dysfunction of cortico-basal ganglia-thalamic networks. Clinical TMS studies most often use figure-of-eight coils to target M1, the supplementary motor area (SMA), or the DLPFC. M1 is the most common motor target. High-frequency rTMS generally uses approximately 5–10 Hz, 90–110% RMT or active motor threshold (AMT), and about 1000–2000 pulses per hemisphere to address bradykinesia, rigidity, and overall motor function measured by the Unified Parkinson’s Disease Rating Scale Part III (UPDRS-III). SMA stimulation more often targets impaired movement initiation, gait dysfunction, and freezing of gait, but both 1 Hz and 10 Hz protocols have been reported, and no standardized parameters have emerged. High-frequency left DLPFC stimulation is used mainly for comorbid depressive symptoms rather than core motor symptoms. TBS is attractive because of its short duration but remains exploratory.

The available evidence provides a comparatively consistent signal for improvement of motor symptoms with high-frequency M1 rTMS. A multicenter randomized trial by Brys et al. (2016) showed that bilateral 10 Hz M1 stimulation improved bradykinesia and rigidity, whereas DLPFC stimulation did not produce a consistent additional antidepressant effect [59]. Song et al. (2024) further reported improvement in freezing of gait and overall motor scores after bilateral 10 Hz M1 stimulation [60]. For the SMA, Mi et al. (2019) found that 10 Hz stimulation improved freezing of gait and motor function [61], whereas Shirota et al. (2013) observed a relatively sustained improvement in UPDRS-III after 1 Hz SMA stimulation [62]; this leaves the optimal direction of SMA modulation uncertain. A systematic review and meta-analysis by Zhang et al. (2022) found small-to-moderate overall effects of rTMS on motor and depressive symptoms, with relatively consistent motor outcomes for high-frequency M1 stimulation [63]. A 2025 meta-analysis by Chen et al. likewise suggested that SMA stimulation may improve selected motor outcomes, although freezing of gait and walking speed were not consistently improved [64]. For TBS, the meta-analysis by Cheng et al. (2022) identified possible benefits only in selected subgroups, with the overall evidence limited by small samples and parameter heterogeneity [65].

TMS in PD is therefore best used as an adjunct to pharmacotherapy and motor rehabilitation rather than as a replacement for levodopa, optimization of dopaminergic medication, or deep brain stimulation. The most reproducible signal is associated with 5–10 Hz rTMS over bilateral M1 or the hemisphere corresponding to the more affected side, particularly for bradykinesia and rigidity. SMA stimulation may be considered when freezing of gait and movement initiation are prominent, but the optimal frequency, dose, and medication state have not been standardized. DLPFC stimulation mainly addresses comorbid depression, with insufficient evidence for consistent cognitive or executive benefits. TBS remains under protocol development. Current limitations include small samples, inconsistent medication ON/OFF states, differences in stimulation parameters and target localization, and short follow-up, so long-term efficacy and patient stratification require further study [63,64,66]. Representative protocols and limitations are summarized in Table 5.

#### 4.2.5. Post-Traumatic Stress Disorder

Post-traumatic stress disorder (PTSD) involves abnormalities in fear processing, traumatic memory, and prefrontal–limbic regulation [68]. Conventional TMS generally uses a figure-of-eight coil over the DLPFC, exploiting high superficial focality to modulate prefrontal influences on emotion, threat monitoring, and cognitive-control networks. Three broad strategies have been studied: 1 Hz low-frequency rTMS over the right DLPFC; 10–20 Hz high-frequency rTMS over the right, left, or bilateral DLPFC; and iTBS over the right DLPFC. Low-frequency protocols often use approximately 90–120% of motor threshold. Pulse dose and treatment duration vary substantially across high-frequency protocols, and no consensus has emerged on laterality or dosing.

Conventional TMS may improve some PTSD symptoms, but results differ across protocols. Randomized sham-controlled studies by Watts et al. (2012) and Leong et al. (2020) reported signals of improvement after 1 Hz right DLPFC stimulation [69,70]. Cohen et al. (2004) and Boggio et al. (2010) explored high-frequency stimulation of the right or left DLPFC, and some studies reported improvement in core PTSD symptoms and associated anxiety or depression [71,72]. Ahmadizadeh et al. (2018) suggested that bilateral 20 Hz stimulation may have therapeutic effects, although a consistent advantage over unilateral stimulation has not been established [73]. Philip et al. (2019) used right DLPFC iTBS and found possible improvements in functional and symptom outcomes; however, core PTSD efficacy was limited during the double-blind phase, and later improvement was difficult to interpret because treatment was open-label [74]. Importantly, a 2024 Cochrane review by Brown et al. judged the number and size of available rTMS studies to be limited and noted that the overall reduction in PTSD severity relative to sham remains uncertain [75].

Figure-of-eight stimulation of the DLPFC remains the main technical approach in conventional TMS research for PTSD, but no single protocol—right-sided low frequency, high frequency, bilateral stimulation, or iTBS—can yet be preferred consistently. Tolerability is generally good; common adverse effects include transient headache, scalp discomfort, fatigue, and treatment-related anxiety. Efficacy is limited by small samples, differences in trauma type and comorbid depression, inconsistent stimulation and localization methods, and short follow-up. Conventional TMS is therefore better positioned as an adjunctive or investigational option when medication or trauma-focused psychotherapy is insufficient, rather than as a replacement for standard care. Because figure-of-eight coils mainly cover the superficial lateral prefrontal cortex and have limited direct access to midline emotion-regulation nodes such as the medial prefrontal cortex (mPFC) and anterior cingulate cortex (ACC), research has also moved toward H-coils and other deep-stimulation technologies. Representative conventional protocols are summarized in Table 6.

## 5. Clinical Applications of Deep Transcranial Magnetic Stimulation

### 5.1. Deep-Coil Design

#### 5.1.1. Double-Cone Coil

**Figure 6:**
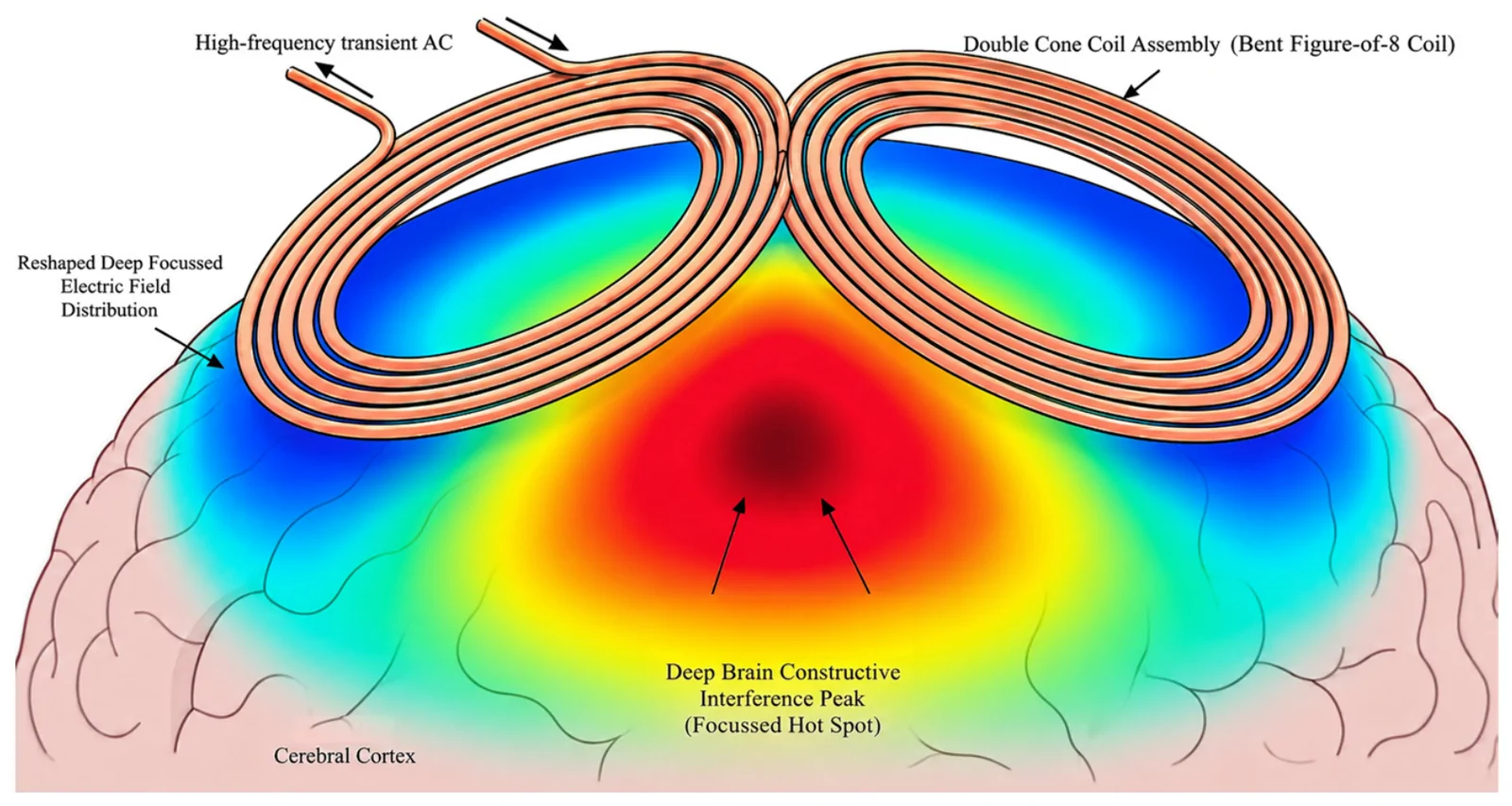
Schematic illustration of the working effect of a double-cone coil.

Conventional figure-of-eight coils provide high superficial focality, but their induced field decays rapidly as the coil-to-cortex distance increases, which limits direct stimulation of medial motor areas and other relatively deep targets. To extend effective deep-field coverage, the double-cone coil places two large circular windings at an angle, which allows the coil to conform more closely to the curvature of the head and alters the spatial superposition of the fields generated by the two wings (Figure 6).

Unlike a planar figure-of-eight coil, the two windings of a double-cone coil are not coplanar but rather form a fixed dihedral angle [10,76]. The traditional double-cone coil has an angle of approximately 95° [10], whereas comparable deep coils such as the MagVenture D-B80 have an angle of approximately 120° [77]. This angled structure both improves conformity to the curved head surface and changes how field vectors from the two wings superimpose, extending the effective central field into deeper tissue [10,19]. Relative to a conventional planar figure-of-eight coil, a double-cone coil therefore tends to show slower decay with depth, a broader superficial field, and lower spatial focality.

Computational models and experimental studies indicate that the effective stimulation depth of a double-cone coil can reach approximately 3–4 cm and may extend further under specific modeling and stimulation conditions [10]. This property makes the coil suitable for relatively deep or midline targets such as the lower-limb motor cortex and medial motor areas. Its engineering advantage is not highly focal stimulation of a single deep structure but rather greater deep-field coverage obtained through controlled spatial spread within the depth–focality trade-off. Similar finite-element investigations of alternative deep-coil designs have demonstrated that improved field penetration is generally accompanied by stimulation of a larger volume of surrounding tissue [78]. The double-cone coil can therefore be regarded as a transitional design between highly focal superficial stimulation and broad deep stimulation.

In addition to double-cone and H-coil architectures, alternative deep-stimulation designs have combined large auxiliary circular or Halo coils with conventional circular coils. Computational studies by Lu and Ueno showed that coaxial Halo–circular assemblies could increase the relative electric-field magnitude in deeper brain regions, although the gain in penetration was generally accompanied by broader spatial exposure and reduced focality [79].

#### 5.1.2. H-Coil

The H-coil is a three-dimensional configuration developed to increase the effective depth and spatial coverage of conventional TMS, with systematic development initiated by Zangen and colleagues in the early 2000s [11]. Unlike the relatively simple planar or angled geometry of circular, figure-of-eight, and double-cone coils, H-coils use multilayer distributed windings that conform to the head. The locations, orientations, and spatial arrangement of multiple conductor elements are adjusted so that their induced fields superimpose in a defined pattern over the target region. Internal winding layouts differ substantially among H-coil models, and some turn counts, conductor dimensions, and routing paths are proprietary and incompletely disclosed. Engineering parameters must therefore be compared model by model.

Modeling studies show that H-coils can generate nonzero electric fields at deeper locations; however, at commonly used safe intensities, the depth of direct suprathreshold activation is usually about 3 cm. Extending farther requires a substantial increase in the superficial cortical field and is consequently constrained for safety reasons [80]. Because greater depth generally enlarges the stimulated volume, the defining feature of an H-coil is not highly focal activation of a single deep structure but rather increased coverage of deeper and broader brain regions and associated networks at the cost of some spatial selectivity. This characteristic again illustrates the depth–focality trade-off and makes H-coils suitable for neuropsychiatric disorders involving multiple cortical and network nodes.

**Figure 7:**
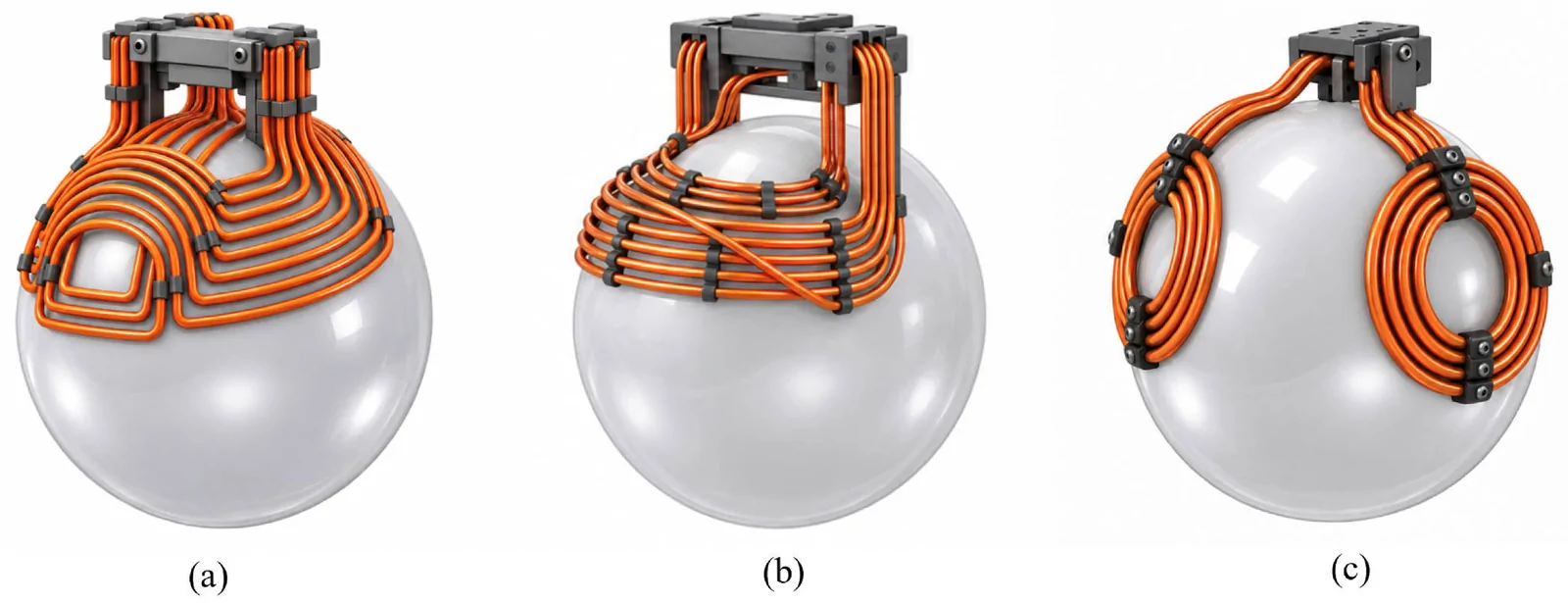
Examples of the external appearance of the (**a**) H1, (**b**) H4, and (**c**) H7 coils (schematic summary based on findings reported in Ref. [81]).

H-coil models alter electric-field coverage by changing the spatial layout of their three-dimensional windings. H1, H4, and H7 are among the configurations with comparatively mature translational evidence, and their representative geometries are shown in Figure 7 [81]. The H1 coil generates a broad prefrontal field with a degree of left-sided predominance and is used mainly to modulate prefrontal networks in MDD [82]. The H4 coil emphasizes bilateral prefrontal and insula-related networks and is used primarily in interventions for nicotine dependence [83]. The H7 coil emphasizes the mPFC and ACC and is used to modulate cortico-striato-thalamo-cortical networks implicated in OCD [84,85]. Although all three belong to the H-coil family, their winding layouts, field distributions, and clinical targets differ; “H-coil” should not be treated as a single configuration with a uniform field pattern. Their geometric features, principal targets, typical parameters, and applications are summarized in Table 7.

Despite the availability of numerous derivative models, the H1, H4, and H7 coils currently represent the principal H-coil configurations that have received approval from the U.S. Food and Drug Administration (FDA) for clinical use, particularly when considering the maturity of clinical translation and evidence-based support. Figure 7 illustrates the geometric configurations of these three representative H-coils. The designs of these coils are closely aligned with the pathological circuit characteristics of their corresponding indications.

The H-coil family also includes H2, H5, H6, H8, H10, H11, and other configurations designed for motor, temporal, and other brain regions. The evidence for these variants is generally more limited, and some remain in preclinical validation or early clinical investigation. This review therefore focuses on the clinically better-established H1, H4, and H7 coils rather than discussing every variant individually.

Table 8 compares the principal technical features of the four representative TMS coil classes discussed in this review, including engineering design, field distribution, and stimulation performance. Because studies differ in stimulator output, current waveform, head model, and tissue-conductivity assumptions, absolute electric-field magnitudes are not directly comparable. Group-level simulations using multiple anatomically realistic head models further demonstrate substantial differences among coil configurations in deep-region electric-field strength, spatial spread, and penetration characteristics [86]. We therefore prioritize measures such as half-value depth, spatial spread, and field-distribution characteristics.

Overall, circular coils provide broad superficial coverage but limited focality; figure-of-eight coils achieve high superficial focality through field superposition at the winding junction; double-cone coils use their angled geometry to extend the axial field deeper; and H-coils use three-dimensional distributed windings to further expand deep-field and network coverage. No coil is universally optimal. Engineering performance reflects a trade-off among depth, focality, and coverage, and coil selection should be based on target anatomy and the intended clinical modulation.

### 5.2. Clinical Translation of Deep Transcranial Magnetic Stimulation

#### 5.2.1. Major Depressive Disorder

As described for conventional TMS, high-frequency rTMS and iTBS delivered with a figure-of-eight coil to the left DLPFC are well-established approaches for MDD. Deep TMS broadens prefrontal electric-field coverage and thereby offers a network-modulation strategy distinct from focal superficial stimulation. H1-coil dTMS remains the deep-stimulation approach with the strongest evidence in MDD; it covers bilateral prefrontal regions with a left DLPFC predominance. Representative parameters are 18 Hz, 120% RMT, and 1980 pulses per session, commonly delivered five times per week for approximately 4 weeks [82,87]. The H7 coil shifts coverage toward the medial prefrontal and anterior cingulate regions and provides an alternative prefrontal-network strategy [85,88].

The principal clinical evidence for the H1 coil comes from the multicenter randomized double-blind sham-controlled trial by Levkovitz et al. (2015) [82]. In the per-protocol analysis, H1 dTMS improved depressive symptoms more than sham, with response rates of 38.4% versus 21.4% and remission rates of 32.6% versus 14.6%, respectively [82,87]. Results were not identical across analysis populations, however, so efficacy estimates remain sensitive to adherence and completion of an adequate treatment course. Subsequent studies extended H1 use to late-life depression, marked anxiety symptoms, and other clinical populations [89,90,91]. For H7, Zangen et al. (2023) compared high-frequency H7 stimulation of the medial prefrontal cortex with H1 stimulation of the lateral prefrontal cortex and supported H7 as another prefrontal-network approach in MDD [88]. Its evidence, however, is derived mainly from active comparison with H1; independent sham-controlled and long-term maintenance evidence remains less extensive [88]. A systematic review and meta-analysis by Lan et al. (2024) generally supported the antidepressant effects of dTMS in treatment-resistant depression but identified heterogeneity in coil configuration, dose, and patient characteristics [92].

For adults with MDD who respond inadequately to antidepressant medication, H1 stimulation at 18 Hz, 120% RMT, and 1980 pulses remains a comparatively well-supported deep TMS protocol [82,87]. H7 provides an alternative that emphasizes the medial prefrontal–anterior cingulate network; the relevant regulatory document is FDA K220819 (2022) [93]. The current evidence is insufficient to conclude that H7 is superior to or has replaced H1. dTMS is generally well tolerated, with headache and stimulation-site discomfort among the most common adverse effects. Major limitations include broad field coverage, difficulty comparing dose across coils, and incomplete standardization of maintenance treatment. Future progress should optimize coil–target matching through individual functional connectivity and electric-field modeling rather than pursuing depth alone. Representative evidence, parameters, and limitations are summarized in Table 9.

#### 5.2.2. Obsessive–Compulsive Disorder

Neuromodulation targets for obsessive–compulsive disorder (OCD) center on the cortico-striato-thalamo-cortical (CSTC) circuit. The medial/dorsomedial prefrontal cortex (mPFC/dmPFC) and dorsal anterior cingulate cortex (dACC) are important cortical nodes for dTMS. The most representative OCD protocol uses an H7 coil to cover bilateral mPFC/dmPFC and dACC. Brief individualized symptom provocation is performed before each session to activate networks related to the patient’s compulsive symptoms. A standard high-frequency protocol uses 20 Hz at 100% of the lower-limb RMT, 2 s trains separated by 20 s, 50 trains, and 2000 pulses per session, for approximately 29 sessions over 6 weeks [84,94,95].

The main evidence comes from randomized trials by Carmi and colleagues. Their preliminary double-blind study in 2018 compared 20 Hz, 1 Hz, and sham stimulation and supported subsequent evaluation of 20 Hz H7 dTMS [94]. Carmi et al. (2019) then conducted a multicenter randomized double-blind sham-controlled trial that combined symptom provocation with 20 Hz H7 stimulation [84]. Active treatment produced greater improvement in Yale–Brown Obsessive–Compulsive Scale (Y-BOCS) scores than sham; response rates were 38.1% and 11.1%, respectively, and a degree of benefit persisted during follow-up [84]. Multicenter real-world data from Roth et al. (2021) further supported feasibility in routine clinical practice [96]. Recent systematic reviews and meta-analyses also indicate a therapeutic signal for TMS in treatment-resistant OCD, although coil type, target, frequency, and study quality remain heterogeneous. Direct randomized evidence is insufficient to establish that H7 is superior to all other configurations [97,98,99].

For adults with persistent moderate-to-severe OCD after adequate serotonin reuptake inhibitor (SRI), exposure and response prevention (ERP), or other standard treatment, symptom provocation combined with 20 Hz H7 dTMS is one of the more standardized and evidence-supported deep-stimulation options. U.S. FDA documentation supports use of the device in adults with OCD [95], but regulatory clearance does not imply routine first-line status in every guideline. The National Institute for Health and Care Excellence (NICE) has found no major safety concern and has judged that the quantity and quality of efficacy evidence remains limited [100]. H7 dTMS is therefore best regarded as a noninvasive adjunctive or optional treatment for refractory OCD, not a replacement for SRIs, ERP, or structured cognitive behavioral therapy. Common adverse effects include transient headache and scalp or facial discomfort, and overall tolerability is good. Representative evidence and limitations are shown in Table 10.

#### 5.2.3. Substance Addiction: Nicotine Dependence

Clinical evidence for dTMS in substance addiction is most concentrated in nicotine dependence. The H4/HADD coil is a representative configuration that covers bilateral prefrontal and insula-related addiction networks [101,102]. A commonly studied protocol uses 10 Hz, 120% of motor threshold, and 1800 pulses per session, delivered five times weekly for the first 3 weeks and once weekly for the next 3 weeks, for approximately 18 sessions in total. Brief smoking-cue or craving induction is usually performed before treatment [83,103]. Outcomes should emphasize biochemically verified continuous abstinence and relapse rather than subjective craving alone in order to determine whether stimulation produces a clinically meaningful cessation effect.

The principal evidence includes both positive findings and an independent negative trial. In an early randomized HADD coil study, Dinur-Klein et al. (2014) found that 10 Hz stimulation combined with smoking-cue provocation reduced smoking behavior and improved selected cessation outcomes [102]. A subsequent multicenter randomized double-blind sham-controlled trial by Zangen et al. (2021) and US Food and Drug Administration (2020) supported an improvement in short-term continuous abstinence with H4 dTMS and formed an important basis for later regulatory use [83,103]. Bellini et al. (2024), however, used a similar H4 protocol—10 Hz, 120% of motor threshold, and 1800 pulses—and found no advantage of active stimulation over sham in 12-week abstinence, cigarette consumption, craving, or biochemical measures [104]. This independent negative result suggests that efficacy may depend on adherence, the intensity of behavioral support, sham response, course completion, and the cue-provocation procedure; stable long-term benefit cannot be inferred from early positive trials alone. A study of H11 stimulation combined with varenicline by Ibrahim et al. (2023) also showed preliminary cessation signals, but the small sample and concomitant medication precluded isolation of the stimulation effect [105].

The current clinical application of dTMS in substance addiction should therefore be restricted primarily to adjunctive smoking-cessation treatment in adults. Relevant FDA documentation supports dTMS delivered with an H4/HADD coil as an aid to short-term smoking cessation in adults [103], but it does not replace varenicline, nicotine-replacement therapy, bupropion, cognitive behavioral interventions, or comprehensive cessation support. Because independent randomized trials have not produced fully consistent results, H4 dTMS is most appropriate for motivated patients who have responded inadequately to conventional treatment and can complete an intensive course, and it should be integrated with behavioral support. Research on alcohol, cocaine, and other substance use disorders remains exploratory, and findings in nicotine dependence cannot be generalized directly to other addictions [106,107,108]. Representative protocols and limitations are presented in Table 11.

#### 5.2.4. Targeted Neuromodulation for Post-Traumatic Stress Disorder

As discussed for conventional TMS, figure-of-eight coils modulate PTSD-related prefrontal–limbic networks mainly through the superficial DLPFC and provide limited direct coverage of midline nodes such as the mPFC and ACC. dTMS studies therefore use H1 or H7 coils to expand electric-field coverage over prefrontal and mPFC/ACC-related networks. Most studies have used high-frequency stimulation combined with brief individualized trauma scripts or memory activation to examine interactions between stimulation state and trauma-related networks. Although H-coils increase depth and coverage, they cannot selectively stimulate the amygdala or hippocampus directly; their effects are more likely mediated indirectly through prefrontal and midline cortical nodes.

The core clinical findings are inconsistent. In a small randomized double-blind sham-controlled study, Isserles et al. (2013) combined an H1 coil with individualized trauma scripts and delivered 20 Hz at 120% of motor threshold, 1680 pulses per session, for 12 sessions [109]. Active stimulation with trauma-memory activation showed a signal of improvement in PTSD symptoms, but only 30 participants were included, and the independent contributions of stimulation, exposure, and their interaction could not be separated [109]. Isserles et al. (2021) subsequently conducted a larger multicenter randomized trial using an H7 coil over bilateral mPFC/ACC at 18 Hz, 100% of the foot RMT, and 2880 pulses per session, together with brief trauma-script exposure [110]. Active H7 stimulation was not superior to sham regarding improvement in the Clinician-Administered PTSD Scale for DSM-5 (CAPS-5), response, or remission [110]. Current evidence therefore does not support the simple assumption that broader and deeper network coverage necessarily produces consistently greater clinical benefit in PTSD.

dTMS for PTSD should currently be regarded as an adjunctive, investigational intervention undergoing protocol optimization. The H1 study provided an initial positive signal, whereas the multicenter H7 trial did not show a clear advantage; this suggests that coil configuration, dose, patient characteristics, and the temporal relationship between trauma-memory activation and stimulation may all affect outcomes [109,110]. Evidence is insufficient to establish either H1 or H7 as a standard deep-stimulation protocol for PTSD or to replace trauma-focused psychotherapy, pharmacotherapy, or comprehensive management. Common safety events include headache, scalp or facial discomfort, and anxiety during stimulation. One generalized tonic–clonic seizure was reported in the H1 pilot study, which underscores the need for rigorous seizure-risk screening before high-frequency H-coil treatment [109]. Representative studies and limitations are summarized in Table 12.

**Figure 8:**
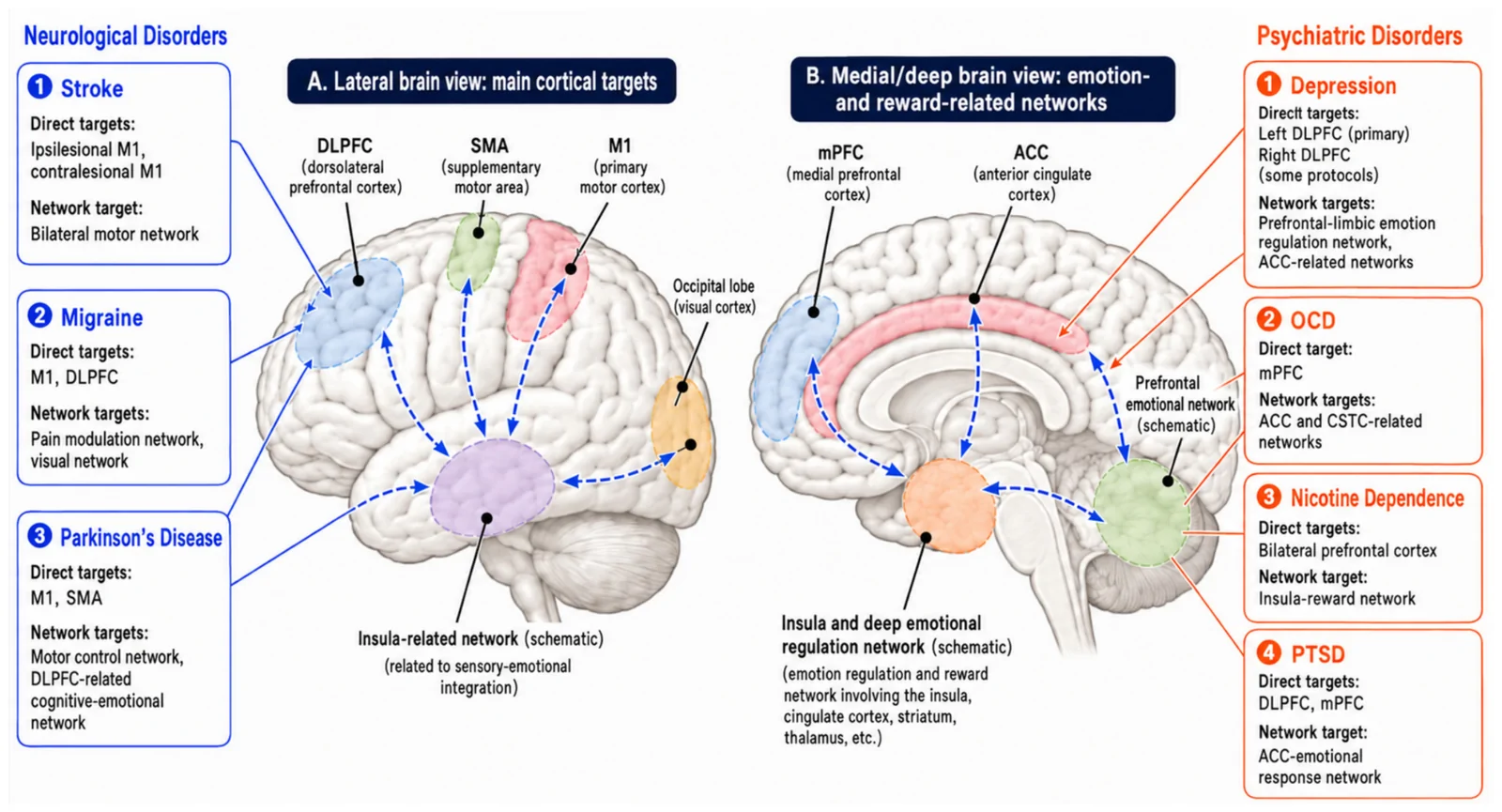
Direct stimulation targets and putative neural networks modulated by transcranial magnetic stimulation in different disorders.

In summary, conventional and deep TMS are not confined to wholly separate disease categories. Clinical selection depends on the anatomical location and spatial distribution of disease-related targets, coil-induced field characteristics, and stimulation parameters. Figure-of-eight coils are suited to superficial, well-defined targets such as the DLPFC, M1, and SMA. Double-cone and H-coils extend effective depth and coverage, providing different strategies for networks involving the mPFC, ACC, and insula. Figure 8 summarizes the principal direct targets and putative networks discussed in this review; Table 13 and Table 14 synthesize representative coils, targets, parameters, evidence, and current clinical positioning in neurological and psychiatric disorders, respectively.

## 6. Safety of Transcranial Magnetic Stimulation

Clinical studies generally indicate that TMS is safe and well tolerated. Most adverse effects are mild and transient and include headache, scalp or local stimulation discomfort, facial or jaw muscle contraction, fatigue, and brief dizziness. The adverse-event profile is broadly similar across disorders and coil types. Headache was among the more common events in the pivotal H7 dTMS trial for OCD [84]. PTSD studies also reported headache, scalp or facial discomfort, local muscle contraction, and fatigue [74,109,110], while some migraine studies observed tinnitus or somnolence [52,53,54,56]. TMS-related discomfort is therefore concentrated in local sensory, auditory, and muscular effects, and there is no evidence of persistent structural neurological injury when treatment is delivered according to established practice.

Seizure is uncommon but remains the most important serious acute risk of TMS; risk is related to frequency, intensity, pulse dose, and individual susceptibility [31]. Isserles et al. (2013) reported one generalized tonic–clonic seizure in an H1 dTMS study for PTSD [109], whereas most standardized clinical trials reported no such events—for example, no seizures occurred in the active arm of the pivotal H7 trial for OCD [84]. Screening and dose control should therefore be strengthened for high-frequency, high-intensity, or high-pulse protocols and for patients with epilepsy, brain injury, or other factors that lower seizure threshold.

Pretreatment assessment should systematically address previous seizures or loss of consciousness, intracranial lesions, recent brain injury, medication use, alcohol or other substance use, and metal or electronic implants in or near the head and neck. FDA device documentation requires hearing protection during treatment and particular attention to ferromagnetic implants, cochlear implants, and implanted neurostimulators near the coil [95]. Active devices such as cochlear implants and deep brain stimulators require individualized risk assessment based on implant location, distance from the coil, manufacturer instructions, and current safety guidance [31]. Protocols involving symptom provocation, such as those used in PTSD, should also monitor anxiety, mood deterioration, and suicidal ideation [110].

Safety management should extend throughout the treatment course, with motor threshold and tolerability reassessed after material changes in stimulation parameters, medication, or clinical status. High-frequency rTMS, TBS, dTMS, and accelerated protocols differ in effective field coverage and cumulative pulse dose; safety parameters should not be extrapolated directly across coils or protocols. International Federation of Clinical Neurophysiology (IFCN) safety guidelines emphasize standardized screening, parameter control, professional training, and risk assessment for novel devices [31]. Treatment centers should maintain standardized adverse-event documentation, seizure-response procedures, and repeated risk assessment, integrating evidence-based treatment guidance [3], device-specific requirements, and individual risk factors.

## 7. Conclusions and Discussion

TMS is a noninvasive neuromodulation technology grounded in electromagnetic induction and neural plasticity. Its biological effects are not determined by a single variable such as intensity or frequency but rather by the combined influence of coil geometry, the intracranial induced electric field, target selection, temporal pulse structure, and individual brain anatomy and functional state. Technically, TMS coils have evolved from circular coils to figure-of-eight coils and then to double-cone and H-coils. This development has not simply pursued greater depth; it has continually balanced stimulation depth, spatial focality, and effective network coverage. The high superficial focality of figure-of-eight coils suits relatively well-defined cortical targets such as the DLPFC and M1. Double-cone and H-coils increase field depth and spatial extent, improving access to medial or relatively deep regions and distributed networks while reducing spatial selectivity. The depth–focality trade-off therefore remains a central framework for understanding the engineering performance and clinical suitability of TMS coils.

Evidence maturity and protocol standardization differ substantially across clinical indications. MDD is among the most thoroughly studied. High-frequency rTMS with a figure-of-eight coil over the left DLPFC has a mature evidence base and corresponding guideline and regulatory support [3]. iTBS substantially shortens sessions by reorganizing the temporal pulse pattern, and the multicenter THREE-D trial established noninferiority to conventional high-frequency rTMS [20]. For deep TMS, high-frequency H1 stimulation of bilateral prefrontal regions with a left DLPFC predominance is supported by a multicenter randomized double-blind sham-controlled trial, although results differed across analysis sets and optimal maintenance remains unresolved [82]. In OCD, 20 Hz H7 dTMS covering the mPFC, ACC, and associated CSTC circuitry has a relatively clear target and parameter framework. The randomized trial by Carmi et al. (2019) and Roth et al. (2021) indicated benefit in some patients with insufficient response to previous treatment, and state-dependent strategies such as symptom provocation may further affect response [84,96]. By contrast, post-stroke motor recovery, migraine, PD, nicotine dependence, and PTSD show therapeutic signals but remain heterogeneous in terms of patient characteristics, disease stage, target, pulse sequence, dose, and outcome assessment. They have not achieved the degree of standardization seen in MDD or OCD. TMS mechanisms and efficacy should therefore not be reduced to a simple high-frequency excitation/low-frequency inhibition model; interpretation should integrate disease-related networks, stimulation location, field direction and dose, temporal pulse structure, and individual neurophysiological state.

Several challenges must be addressed before TMS can become more precise and standardized. First, identical stimulator output does not produce an identical effective target dose in every individual. Realistic head modeling by Laakso et al. (2014) showed that gyral and sulcal morphology, tissue conductivity, head curvature, and scalp-to-cortex distance can substantially alter field magnitude, direction, and peak location [5]. Dosing based on motor threshold and percentage of maximum stimulator output is clinically convenient but does not fully represent the electric-field dose received by brain tissue. Second, fixed anatomical targets do not capture the network organization of neuropsychiatric disorders, and individual differences in functional connectivity, lesion location, structural integrity, and baseline brain state may modify response. Third, novel coils, dTMS, TBS, and accelerated protocols differ substantially in field extent and per-session and cumulative pulse dose, so their safety parameters cannot be transferred directly across devices and protocols. Future standardization must therefore go beyond frequency, intensity, and pulse count to an integrated dosimetry framework that describes coil configuration, spatial field dose, temporal dose, and patient state while incorporating standardized screening, repeated risk assessment, and device-specific safety requirements throughout treatment [31].

Future TMS development may shift from maximizing depth or focality alone toward systematic matching among coil configuration, intracranial electric field, disease network, pulse sequence, and individual brain state. Multichannel coil arrays provide another engineering route toward flexible field control: by adjusting the current direction and relative phase across multiple small coils, the magnitude and spatial distribution of the induced electric field can be modified without mechanically repositioning a single conventional coil [111]. Finite-element calculations using realistic head models and individualized field simulations can optimize coil position, orientation, and dose and reduce reliance on empirical percentages of device output. Structural magnetic resonance imaging (MRI), functional MRI, and neuronavigation can improve target localization and move stimulation from fixed scalp landmarks toward individualized disease-network targets [112]. Multimodal methods such as TMS–electroencephalography (TMS-EEG) can characterize cortical and network responses after stimulation and provide objective neurophysiological information for target selection, response prediction, and parameter adjustment. Clinical translation of novel coils, accelerated stimulation, and high-dose protocols still requires large multicenter randomized trials and long-term follow-up to establish durability, safety, optimal dose ranges, and the populations most likely to benefit. The key question is no longer whether a coil can “stimulate deeper,” but rather whether its field can cover a disease-related network with an appropriate spatial extent and dose and produce clinically meaningful, reproducible neuromodulation.

This review integrates conventional and deep TMS across coil design, intracranial induced fields, targets, pulse parameters, clinical efficacy, and safe implementation, establishing an integrated analytical framework from physical design to clinical effect. The framework clarifies the technical advantages, boundaries, and clinical suitability of different coil configurations. It may help researchers working on coil design, electric-field modeling, and precision neuromodulation understand the relationship between engineering parameters and biological effects, and it may support clinicians in coil selection, target definition, treatment planning, and safe delivery. Integrating engineering performance with disease networks and clinical requirements may narrow the translational gap among coil design, computational modeling, and clinical practice and provide a theoretical basis for developing new devices and individualized treatment strategies.

This review has several limitations. First, studies use different head models, tissue conductivities, electric-field thresholds, stimulator outputs, and definitions of depth and focality; reported field strengths and effective depths therefore cannot be compared quantitatively without qualification. Second, engineering information for some commercial H-coils—including turn count, conductor cross-section, internal geometry, material properties, and operating current—has not been fully disclosed in peer-reviewed publications or regulatory documents, which limits independent replication and quantitative comparison. Third, clinical indications differ substantially in terms of coil type, target localization, dose, medication state, concomitant treatment, and follow-up. This review uses narrative synthesis and does not include an independent meta-analysis; raw efficacy estimates across studies should not be used to rank coils or protocols directly. Finally, evidence for some novel coils, individualized field models, network-guided targets, and accelerated protocols comes mainly from small, single-center, or exploratory studies. Long-term safety, durability, and cross-center reproducibility remain uncertain. Future work should standardize coil-performance metrics and TMS dose descriptions, strengthen the mapping among engineering parameters, modeled fields, and clinical outcomes, and test whether individualized electric-field and network-targeting strategies translate into stable clinical benefit in standardized prospective multicenter studies.

Overall, TMS is moving from regional stimulation based on a fixed coil, fixed anatomical target, and empirical parameters toward precision neuromodulation centered on individualized electric-field dose and disease-related neural networks. Coil design remains the physical basis of spatial stimulation characteristics, but future progress will depend less on maximizing any single performance metric than on coordinating engineering design, field dose, network properties, temporal stimulation pattern, and patient state. A unified, quantitative, and clinically interpretable framework for TMS dosing and targeting will be essential to improve reproducibility, optimize patient selection, and advance the clinical translation of emerging TMS technologies.

## Figures and Tables

**Table 1:** Summary of Different Stimulation Frequencies and Their Effects on Neuroplasticity.

Stimulation Mode	Typical Frequency/Pattern	General Modulatory Tendency	Typical Applications
High-frequency rTMS	≥5 Hz; commonly 10–20 Hz	Facilitatory	Left dorsolateral prefrontal cortex (DLPFC); primary motor cortex (M1)
Low-frequency rTMS	≤1 Hz	Inhibitory	Right DLPFC; contralesional M1
iTBS	50-Hz triplet bursts repeated at 5 Hz	Predominantly facilitatory	Depression; motor networks
cTBS	Continuous TBS	Predominantly inhibitory	Motor-cortex/network modulation

**Table 2:** Key parameters and clinical efficacy of representative stimulation protocols described above and their clinical efficacy reported in representative high-quality studies.

Protocol and Representative Evidence	Coil and Target	Core Parameters	Main Clinical Findings	Main Limitations
Left-sided 10 Hz rTMS; O’Reardon et al. [29]	Figure-of-eight; left DLPFC	10 Hz; 120% RMT; ~3000 pulses/session; 4–6 weeks	Mature evidence base; active stimulation superior to sham	Long sessions; interindividual variability in response
Right-sided low-frequency rTMS [32,33,34]	Figure-of-eight; right DLPFC	1 Hz; 110–120% RMT; ~600–1600 pulses/session	Improves depressive symptoms; some studies show efficacy similar to left-sided high-frequency rTMS	Substantial heterogeneity in parameters and patient selection
Standard iTBS; Blumberger et al. [20]	Figure-of-eight; left DLPFC	50 Hz triplet bursts at 5 Hz; 120% RMT; 600 pulses; ~3 min	Noninferior to 10 Hz rTMS and markedly shorter	Not superior to conventional rTMS for every patient
Accelerated individualized iTBS; Cole et al. [36]	Figure-of-eight; connectivity-guided left DLPFC	1800 pulses/session; multiple daily sessions; ~5 days	Early studies show rapid, high response signals	High dose and resource demand; independent validation required

**Table 3:** Comparison of common transcranial magnetic stimulation protocols for post-stroke motor dysfunction.

Protocol and Representative Studies	Coil and Target	Core Parameters	Main Efficacy Findings	Main Limitations
Contralesional low-frequency rTMS; Luk et al., 2022 [42]; Kim et al., 2020 [46]; Qian et al., 2024 [47]	Figure-of-eight; contralesional M1	1 Hz; 80–100% RMT; ~900–1800 pulses; 10–15 sessions	May improve the Fugl-Meyer Assessment-Upper Extremity (FMA-UE) score and hand function; patients with MEPs or a preserved corticospinal tract (CST) may benefit more	Marked heterogeneity; affected by lesion location and structural reserve
Ipsilesional high-frequency rTMS; Chang et al., 2010 [48]	Figure-of-eight/butterfly; ipsilesional M1	5–20 Hz; 80–100% RMT; ~1000–1500 pulses	May increase ipsilesional excitability and improve motor function	Parameters and course not standardized
Contralesional cTBS; Kuzu et al., 2021 [43]; Vink et al., 2023 [44]	Figure-of-eight; contralesional M1	50 Hz triplet bursts repeated continuously at 5 Hz; 600 pulses	Some studies show improvement in ARAT and upper-limb motor function	Limited evidence and substantial patient heterogeneity
Ipsilesional iTBS; Chen et al., 2019 [45]; Kim et al., 2015 [49]	Figure-of-eight; ipsilesional M1	50 Hz triplet bursts repeated intermittently at 5 Hz; 600 pulses	May improve FMA-UE, fine motor function, and selected spasticity measures	Mostly small studies; larger randomized controlled trials (RCTs) are required

**Table 4:** Comparison of different transcranial magnetic stimulation paradigms for migraine.

Protocol and Representative Studies	Primary Target	Representative Parameters and Coil	Main Efficacy Findings	Main Limitations
High-frequency M1 rTMS; Misra et al., 2013 [52]; Shehata et al., 2016 [53]	Left M1	Figure-of-eight; 10 Hz; 70–80% motor threshold (MT); ~600–2000 pulses/session	May reduce attack frequency and selected pain outcomes	Substantial variation in parameters and treatment courses
High-frequency DLPFC rTMS; Brighina et al., 2004 [54]; Conforto et al., 2014 [55]	Left DLPFC	Figure-of-eight; ~5–20 Hz; several hundred to ~2000 pulses/session	Some studies show lower attack frequency, but results are inconsistent	Small samples and substantial placebo and parameter heterogeneity
iTBS; Sahu et al., 2019 [56]	Left DLPFC	Figure-of-eight; 50 Hz triplet bursts at 5 Hz; 80% AMT; ~600 pulses	Preliminary improvement in attack frequency, duration, and pain	Single-center small study; no replication
Systematic reviews/meta-analyses; Moisset et al., 2020 [57]; Zhong et al., 2022 [58]	M1, DLPFC, and others	Predominantly high-frequency rTMS	May reduce attack frequency and headache days overall	No consensus on optimal target, pain-intensity benefit, or parameters

**Table 5:** Comparison of TMS protocols for Parkinson’s disease.

Protocol and Representative Studies	Primary Target	Representative Parameters and Coil	Main Efficacy Findings	Main Limitations
High-frequency M1 rTMS; Brys et al., 2016 [59]; Song et al., 2024 [60]	Bilateral M1 or M1 contralateral to the more affected side	Figure-of-eight; 10 Hz; ~90–110% MT; ~1000 pulses/side	Improvement signals for bradykinesia, rigidity, and selected freezing-of-gait outcomes	Effects are mainly short term; axial-symptom results are inconsistent
Low-frequency SMA rTMS; Shirota et al., 2013 [62]	SMA	Figure-of-eight; 1 Hz; 110% AMT; 1000 pulses/session	May improve UPDRS-III	Unusual treatment schedule limits direct extrapolation to daily practice
High-frequency SMA rTMS; Mi et al., 2019 [61]	SMA	Figure-of-eight; 10 Hz; ~1000 pulses/session	May improve freezing of gait and overall motor function	Small sample; optimal frequency undetermined
DLPFC rTMS; Cardoso et al., 2008 [67]; Brys et al., 2016 [59]	Left DLPFC	Figure-of-eight; 5–10 Hz	Used mainly for comorbid depressive symptoms; results are inconsistent	Should not be extrapolated as a protocol for motor or cognitive improvement
TBS; Cheng et al., 2022 [65]	SMA, M1, and others	iTBS/cTBS; heterogeneous parameters	Selected subgroups may show motor improvement	Few studies; no standard prescription

**Table 6:** Representative studies of planar coils and stimulation protocols for post-traumatic stress disorder.

Protocol and Representative Studies	Primary Target	Representative Parameters and Coil	Main Efficacy Findings	Main Limitations
Low-frequency rTMS; Watts et al., 2012 [69]; Leong et al., 2020 [70]	Right DLPFC	Figure-of-eight; 1 Hz; 90–120% MT; ~400–2250 pulses/session	Some studies show improvement in PTSD symptoms	Small samples, large dose variation, and uncertain long-term effects
High-frequency rTMS; Cohen et al., 2004 [71]; Boggio et al., 2010 [72]; Leong et al., 2020 [70]	Right or left DLPFC	Figure-of-eight; 10–20 Hz; 80–120% MT; ~1600–3000 pulses/session	Some studies show improvement in core and associated emotional symptoms	Marked heterogeneity in laterality and dose; inconsistent findings
Bilateral high-frequency rTMS; Ahmadizadeh et al., 2018 [73]	Bilateral DLPFC	Figure-of-eight; 20 Hz; ~2400 pulses/session	Both bilateral and right-sided stimulation showed positive signals	Bilateral stimulation has not shown consistent superiority
iTBS; Philip et al., 2019 [74]	Right DLPFC	Figure-of-eight; iTBS; 80% MT; 1800 pulses/session	Possible improvement in functional and PTSD outcomes	Limited double-blind efficacy; open-phase findings are difficult to interpret
Overall evidence; Brown et al., 2024 [75]	Mainly DLPFC	Multiple rTMS protocols	Potential overall efficacy signal	Few studies, high heterogeneity, and limited certainty

**Table 7:** Design characteristics and principal clinical applications of representative H-coils.

Coil Model	Principal Geometric/Electric-Field Features	Principal Coverage	Typical Parameters	Representative Clinical Applications
H1	Broad prefrontal distribution with a degree of left-sided predominance	DLPFC and related prefrontal networks	Commonly 18 Hz at ~120% RMT	MDD
H4	Broad bilateral prefrontal and insula-related coverage	Prefrontal cortex and insula-related networks	Commonly 10 Hz at ~120% MT	Nicotine dependence
H7	Greater emphasis on medial prefrontal and anterior cingulate regions	mPFC and ACC	Commonly 20 Hz for OCD or 18 Hz in some MDD studies	OCD; MDD research

**Table 8:** Engineering parameters and electromagnetic performance of representative TMS coils.

Coil Type	Typical Geometry/Size	Winding and Conductor Parameters	Current/Pulse Characteristics	Typical Placement and Orientation	Induced Electric-Field Distribution	Typical Effective Depth	Spatial Focality	Current Principal Uses
Circular coil	Single planar circular winding	Wound copper conductor; turns and wire diameter vary by device, and some early studies did not report them completely	Barker’s early system: peak current ~4000 A; pulse duration ~110 μs [1]	Coil plane close to and approximately parallel with the scalp; maximal field near the winding projection rather than the geometric center	Annular broad distribution with a low-field center; tangential spread area up to ~34 cm^2^ [4]	Half-value depth ~1.0–3.5 cm [4,25]	Low	Early cortical stimulation; central motor conduction time; peripheral nerve and nerve-root assessment
Figure-of-eight coil	Two adjacent circular windings with opposite current directions; common clinical configuration ~70 mm	Symmetric windings; turns, wire diameter, and conductor cross-section vary by manufacturer and model	Stimulator output and peak current depend on device and protocol	Winding junction aligned with the target; rotation determines the direction of the central induced current and materially affects the stimulated region	Fields from the two windings superimpose at the junction to form a focal hotspot and decay rapidly away from it [9,19]	Effective stimulation concentrated in superficial cortex, typically ~1.5–2 cm [4]	High	Superficial, well-defined targets including DLPFC, M1, and SMA
Double-cone coil	Two large circular windings folded at a fixed dihedral angle; traditional angle ~95° [10], D-B80 ~120° [77]	Large two-wing winding; turns and wire diameter depend on the model and are not consistently reported	Device- and model-dependent; the source literature does not report directly comparable peak currents	Wings conform to head curvature; central junction generally aligned with a midline or relatively deep target	Field vectors from the two wings superimpose axially, extending deeper while increasing superficial coverage [10,19]	~3–4 cm and sometimes deeper under specific conditions [10]	Moderate to low	Lower-limb motor cortex, medial motor areas, and relatively deep midline targets
H-coil	Helmet-like three-dimensional multilayer distributed windings; spatial layout varies by model	Multiple conductors in multilayer 3D geometry; some turn counts, conductor dimensions, and internal layouts are proprietary and incompletely disclosed	Model- and stimulator-dependent; absolute peak currents are generally not comparable across H-coils	Helmet positioned according to the model to cover designated regions; stimulation direction is determined jointly by the distributed internal windings	Spatial superposition from multiple field sources creates deeper and broader effective coverage, with a much larger focal volume than a figure-of-eight coil [11,80]	No single fixed value; direct activation depth ~3 cm at commonly used safe intensities, with further depth limited by superficial safety [80]	Low, but broad network coverage	MDD, OCD, nicotine dependence, and other indications requiring broad network modulation

Note: Studies use different stimulator outputs, current waveforms, coil-to-scalp distances, head models, tissue conductivities, and electric-field threshold definitions. Absolute induced-field magnitudes (V/m) reported in different publications should therefore not be compared directly. This review prioritizes geometric parameters, half-value depth, spatial spread, and field-distribution features that are relatively stable and comparable within the original studies. Turn count, conductor cross-section, and internal winding parameters that were not disclosed in the original study or by the manufacturer are reported as not reported or not publicly disclosed and are not inferred.

**Table 9:** Comparison of transcranial magnetic stimulation protocols for major depressive disorder.

Protocol and Representative Studies	Principal Coverage	Representative Parameters	Main Evidence	Main Limitations
Standard H1 dTMS; Levkovitz et al., 2015 [82]; FDA K122288 [87]	Bilateral prefrontal cortex with left DLPFC predominance	H1; 18 Hz; 120% RMT; 1980 pulses/session; ~5 sessions/week for 4 weeks	Response 38.4% vs. 21.4%; remission 32.6% vs. 14.6%	Results differ across analysis sets; maintenance protocols are not standardized
High-dose H1 in special populations; Kaster et al., 2018 [89]	Prefrontal cortex with left-sided predominance	H1; high frequency; pulse dose above the standard protocol	Efficacy signal in late-life depression	High-dose parameters should not be adopted directly as routine standards
H1 vs. figure-of-eight; Filipčić et al., 2019 [90]	H1: broad prefrontal cortex; figure-of-eight: left DLPFC	Standard high-frequency H1 protocol vs. conventional high-frequency figure-of-eight rTMS	Both protocols produced antidepressant effects	Active comparison without sham; differences cannot be attributed solely to depth
H7 dTMS; Zangen et al., 2023 [88]; FDA K220819 [93]	mPFC/ACC-related prefrontal networks	H7; high-frequency protocol	Supports H7 as an alternative deep prefrontal protocol for MDD	Less independent sham-controlled and long-term evidence than H1
Overall dTMS evidence; Lan et al., 2024 [92]	Prefrontal networks	Predominantly high-frequency H-coil stimulation	Meta-analysis supports overall symptom improvement in treatment-resistant depression	Heterogeneity in coils, dose, and patient characteristics

**Table 10:** Comparison of deep transcranial magnetic stimulation protocols for obsessive–compulsive disorder.

Protocol and Representative Studies	Principal Target	Representative Parameters	Main Evidence	Main Limitations
Preliminary H7 dTMS protocol; Carmi et al., 2018 [94]	Bilateral mPFC/ACC	20 Hz; 100% lower-limb RMT; 2000 pulses; symptom provocation before treatment	20 Hz group showed a more marked Y-BOCS improvement signal, supporting the subsequent standard protocol	Small sample; also compared 1 Hz and sham
Standard H7 dTMS; Carmi et al., 2019 [84]	Bilateral mPFC/dmPFC and dACC	20 Hz; 100% lower-limb RMT; 2000 pulses; ~29 sessions/6 weeks; symptom provocation	Response 38.1% vs. 11.1%, with a follow-up efficacy signal	Broad stimulation field; maintenance strategy remains unclear
Real-world use; Roth et al., 2021 [96]	mPFC/ACC-related networks	Standard clinical H7 protocol	Supports feasibility and an efficacy signal in routine practice	Nonrandomized real-world data with selection and follow-up bias
Integrated evidence; Li et al., 2024 [97]; Grassi et al., 2023 [98]; Kar et al., 2024 [99]	mPFC/ACC, DLPFC, SMA/pre-supplementary motor area (pre-SMA), and others	Multiple TMS/dTMS protocols	Overall support for improvement in OCD symptoms	Marked heterogeneity in targets, coils, parameters, and study quality

**Table 11:** Comparison of deep transcranial magnetic stimulation studies for nicotine dependence.

Protocol and Representative Studies	Principal Coverage	Representative Parameters	Main Evidence	Main Limitations
Early HADD dTMS protocol; Dinur-Klein et al., 2014 [102]	Bilateral prefrontal and insula-related networks	10 Hz; 120% RMT; 990 pulses; cue provocation	High-frequency stimulation showed signals of reduced smoking and improved selected cessation outcomes	Small samples/subgroups, high attrition, and uncertain durability
Pivotal H4 dTMS protocol; Zangen et al., 2021 [83]; FDA K200957 [103]	Bilateral prefrontal and insula-related networks	10 Hz; 120% MT; 1800 pulses; 3-week intensive phase plus 3-week maintenance; cue provocation	Multicenter RCT supports increased short-term continuous abstinence	Long-term maintenance and patient selection require clarification
Independent H4 dTMS validation; Bellini et al., 2024 [104]	Bilateral prefrontal and insula-related networks	10 Hz; 120% MT; 1800 pulses; up to 21 sessions	Active stimulation was not superior to sham for abstinence, smoking amount, or craving	Single center, high attrition, mid-course protocol adjustment; limited reproducibility
Exploratory H11 protocol; Ibrahim et al., 2023 [105]	Bilateral insular and frontotemporal networks	10 Hz; 120% MT; 1020 pulses; combined with varenicline	Cessation signals at selected time points	Small sample and concomitant medication prevent isolation of stimulation effects

**Table 12:** Representative studies comparing coils and stimulation protocols for post-traumatic stress disorder.

Protocol and Representative Studies	Principal Coverage	Representative Parameters	Main Evidence	Main Limitations
H1 dTMS plus trauma script; Isserles et al., 2013 [109]	Bilateral prefrontal cortex, partial mPFC coverage, left-sided predominance	20 Hz; 120% MT; 1680 pulses/session; 12 sessions/4 weeks	Signals of improvement in PTSD and selected intrusion symptoms	Only 30 participants; limited overall between-group differences; stimulation and exposure effects cannot be separated; one seizure reported
H7 dTMS plus trauma exposure; Isserles et al., 2021 [110]	Bilateral mPFC/ACC	18 Hz; 100% foot RMT; 2880 pulses/session; 12 sessions/4 weeks with intensified stimulation	No superiority over sham for CAPS-5, response, or remission	No demonstrated augmentation; timing of stimulation and trauma exposure may have affected results

**Table 13:** Representative TMS protocols and current clinical positioning in neurological disorders.

Disorder	Current Principal Protocols and Representative Evidence	Coil and Principal Target	Representative Parameters	Core Efficacy Information	Main Limitations	Current Clinical Positioning
Parkinson’s disease (PD)	High-frequency rTMS over M1 or SMA; Brys et al., 2016 [59]; Song et al., 2024 [60]; Mi et al., 2019 [61]	Figure-of-eight; M1 or SMA; DLPFC may be considered for comorbid depression	Usually 5–10 Hz; ~90–110% MT; ~1000–2000 pulses/session	M1 stimulation shows improvement signals for bradykinesia and rigidity; SMA may improve gait/freezing of gait	Small samples; major differences in medication state, target, and course; uncertain long-term effects	Adjunctive/investigational neuromodulation alongside medication and motor rehabilitation
Post-stroke motor dysfunction	Contralesional M1 low-frequency rTMS, ipsilesional M1 high-frequency rTMS, and iTBS/cTBS; Luk et al., 2022 [42]; Chang et al., 2010 [48]; Kuzu et al., 2021 [43]; Vink et al., 2023 [44]; Chen et al., 2019 [45]	Figure-of-eight; contralesional or ipsilesional M1	1 Hz, ~900–1800 pulses; high-frequency protocols generally 5–20 Hz; iTBS/cTBS commonly 600 pulses	Some studies show improvement in Fugl-Meyer Assessment (FMA), ARAT, and upper-limb function	Effects depend on time after stroke, lesion location, MEP status, and CST integrity; large trials are not fully consistent	Adjunctive neuromodulation combined with conventional rehabilitation
Migraine	Preventive high-frequency rTMS/iTBS over M1 or DLPFC; Misra et al., 2013 [52]; Shehata et al., 2016 [53]; Sahu et al., 2019 [56]; meta-analyses [57,58]	Figure-of-eight; left M1 or left DLPFC	M1 commonly 10 Hz, 70–80% MT, 600–2000 pulses; iTBS ~600 pulses	May reduce attack frequency and headache days overall, but improvement in pain intensity is inconsistent	Optimal target, dose, and course are not standardized	Preventive rTMS is mainly adjunctive/investigational; acute single-pulse TMS has a separate evidence base and is outside the scope of this review

**Table 14:** Representative TMS protocols and current clinical positioning in psychiatric disorders.

Disorder	Current Principal Protocols and Representative Evidence	Coil and Principal Target	Representative Parameters	Core Efficacy Information	Main Limitations	Current Clinical Positioning
Major depressive disorder (MDD)	High-frequency rTMS and iTBS over left DLPFC and H1 dTMS; O’Reardon et al., 2007 [29]; Blumberger et al., 2018 [20]; Levkovitz et al., 2015 [82]	Figure-of-eight: left DLPFC; H1: bilateral prefrontal cortex with left-sided predominance; H7 can cover mPFC/ACC-related networks	10 Hz at 120% RMT, ~3000 pulses; iTBS 600 pulses; H1 commonly 18 Hz at 120% RMT, 1980 pulses	Conventional high-frequency rTMS and iTBS have mature evidence; a multicenter H1 RCT supports antidepressant efficacy [82,87]	Marked interindividual variability; maintenance and network-targeting protocols require optimization	One of the most evidence-supported clinical TMS applications reviewed here
Obsessive–compulsive disorder (OCD)	Symptom provocation combined with H7 dTMS; Carmi et al., 2019 [84]	H7; bilateral mPFC/dmPFC and dACC	20 Hz; 100% lower-limb RMT; 2000 pulses/session; ~29 sessions/6 weeks	Pivotal RCT response 38.1% vs. 11.1% [84]	Requires symptom provocation; limited evidence on maintenance and direct comparison with other coils	Adjunctive/optional treatment for treatment-resistant adult OCD; does not replace SRI or ERP
Post-traumatic stress disorder (PTSD)	Conventional low-/high-frequency DLPFC rTMS or iTBS; exploratory deep H1/H7 protocols; Watts et al., 2012 [69]; Philip et al., 2019 [74]; Isserles et al., 2013 [109] and 2021 [110]	Figure-of-eight: DLPFC; H1/H7: prefrontal and mPFC/ACC-related networks	1 Hz or 10–20 Hz; iTBS; predominantly high-frequency H1/H7 protocols	Conventional DLPFC studies show efficacy signals; a small H1 study was positive, whereas a multicenter H7 RCT was not superior to sham	Optimal laterality, frequency, timing of trauma exposure, and coil protocol are not standardized	Potentially beneficial but currently adjunctive/investigational and under protocol optimization
Nicotine dependence	High-frequency H4/HADD dTMS with smoking cues; Dinur-Klein et al., 2014 [102]; Zangen et al., 2021 [83]; Bellini et al., 2024 [104]	H4/HADD; bilateral prefrontal and insula-related networks	10 Hz; 120% MT; 1800 pulses; 3-week intensive phase plus 3-week maintenance	A pivotal multicenter RCT supports short-term cessation, but an independent RCT did not replicate a clear advantage	Long-term efficacy, patient selection, and behavioral-intervention effects remain uncertain	One adjunctive cessation option for adult smokers; findings cannot be generalized to all substance use disorders

## Data Availability

No new data were created or analyzed in this study. Data sharing is not applicable to this article.

## Abbreviations

The following abbreviations are used in this manuscript:

TMS	Transcranial Magnetic Stimulation
rTMS	Repetitive Transcranial Magnetic Stimulation
dTMS	Deep Transcranial Magnetic Stimulation
TBS	Theta-Burst Stimulation
iTBS	Intermittent Theta-Burst Stimulation
cTBS	Continuous Theta-Burst Stimulation
TMS-EEG	Transcranial Magnetic Stimulation-Electroencephalography
DLPFC	Dorsolateral Prefrontal Cortex
mPFC	Medial Prefrontal Cortex
dmPFC	Dorsomedial Prefrontal Cortex
M1	Primary Motor Cortex
SMA	Supplementary Motor Area
pre-SMA	Pre-Supplementary Motor Area
ACC	Anterior Cingulate Cortex
dACC	Dorsal Anterior Cingulate Cortex
CST	Corticospinal Tract
CSTC	Cortico-Striato-Thalamo-Cortical Circuit
MRI	Magnetic Resonance Imaging
MEP/MEPs	Motor-Evoked Potential(s)
MT	Motor Threshold
RMT	Resting Motor Threshold
AMT	Active Motor Threshold
MDD	Major Depressive Disorder
OCD	Obsessive–Compulsive Disorder
PTSD	Post-Traumatic Stress Disorder
PD	Parkinson’s Disease
RCT	Randomized Controlled Trial
FDA	Food and Drug Administration
NICE	National Institute for Health and Care Excellence
IFCN	International Federation of Clinical Neurophysiology
SRI	Serotonin Reuptake Inhibitor
SAINT	Stanford Accelerated Intelligent Neuromodulation Therapy
SNT	Stanford Neuromodulation Therapy
ARAT	Action Research Arm Test
FMA	Fugl-Meyer Assessment
FMA-UE	Fugl-Meyer Assessment-Upper Extremity
UPDRS-III	Unified Parkinson’s Disease Rating Scale, Part III
CAPS-5	Clinician-Administered PTSD Scale for DSM-5
Y-BOCS	Yale–Brown Obsessive–Compulsive Scale
ERP	Exposure and Response Prevention
CSD	Cortical Spreading Depression

## References

[1] Barker A.T., Jalinous R., Freeston I.L. (1985). Non-invasive magnetic stimulation of human motor cortex. Lancet.

[2] Huang Y.Z., Edwards M.J., Rounis E., Bhatia K.P., Rothwell J.C. (2005). Theta burst stimulation of the human motor cortex. Neuron.

[3] Lefaucheur J.P., Aleman A., Baeken C., Benninger D.H., Brunelin J., Di Lazzaro V., Filipović S.R., Grefkes C., Hasan A., Hummel F.C. (2020). Evidence-based guidelines on the therapeutic use of repetitive transcranial magnetic stimulation (rTMS): An update (2014–2018). Clin. Neurophysiol..

[4] Deng Z.D., Lisanby S.H., Peterchev A.V. (2013). Electric field depth–focality tradeoff in transcranial magnetic stimulation: Simulation comparison of 50 coil designs. Brain Stimul..

[5] Laakso I., Hirata A., Ugawa Y. (2014). Effects of coil orientation on the electric field induced by TMS over the hand motor area. Phys. Med. Biol..

[6] Gomez-Tames J., Laakso I., Hirata A. (2020). Review on biophysical modelling and simulation studies for transcranial magnetic stimulation. Phys. Med. Biol..

[7] Guo Q., Li C., Wang J. (2017). Updated review on the clinical use of repetitive transcranial magnetic stimulation in psychiatric disorders. Neurosci. Bull..

[8] Roth Y., Amir A., Levkovitz Y., Zangen A. (2007). Three-dimensional distribution of the electric field induced in the brain by transcranial magnetic stimulation using figure-8 and deep H-coils. J. Clin. Neurophysiol..

[9] Ueno S., Tashiro T., Harada K. (1988). Localized stimulation of neural tissues in the brain by means of a paired configuration of time-varying magnetic fields. J. Appl. Phys..

[10] Lu M., Ueno S. (2017). Comparison of the induced fields using different coil configurations during deep transcranial magnetic stimulation. PLoS ONE.

[11] Roth Y., Zangen A., Hallett M. (2002). A coil design for transcranial magnetic stimulation of deep brain regions. J. Clin. Neurophysiol..

[12] Kujirai T., Caramia M.D., Rothwell J.C., Day B.L., Thompson P.D., Ferbert A., Wroe S., Asselman P., Marsden C.D. (1993). Corticocortical inhibition in human motor cortex. J. Physiol..

[13] Pascual-Leone A., Valls-Solé J., Wassermann E.M., Hallett M. (1994). Responses to rapid-rate transcranial magnetic stimulation of the human motor cortex. Brain.

[14] Pascual-Leone A., Rubio B., Pallardó F., Catalá M.D. (1996). Rapid-rate transcranial magnetic stimulation of left dorsolateral prefrontal cortex in drug-resistant depression. Lancet.

[15] Cohen S.L., Bikson M., Badran B.W., George M.S. (2022). A visual and narrative timeline of US FDA milestones for Transcranial Magnetic Stimulation (TMS) devices. Brain Stimul..

[16] Ugawa Y., Uesaka Y., Terao Y., Hanajima R., Kanazawa I. (1994). Magnetic stimulation of corticospinal pathways at the foramen magnum level in humans. Ann. Neurol..

[17] Zangen A., Roth Y., Voller B., Hallett M. (2005). Transcranial magnetic stimulation of deep brain regions: Evidence for efficacy of the H-coil. Clin. Neurophysiol..

[18] Fox M.D., Buckner R.L., White M.P., Greicius M.D., Pascual-Leone A. (2012). Efficacy of transcranial magnetic stimulation targets for depression is related to intrinsic functional connectivity with the subgenual cingulate. Biol. Psychiatry.

[19] Ueno S., Sekino M. (2021). Figure-eight coils for magnetic stimulation: From focal stimulation to deep stimulation. Front. Hum. Neurosci..

[20] Blumberger D.M., Vila-Rodriguez F., Thorpe K.E., Feffer K., Noda Y., Giacobbe P., Knyahnytska Y., Kennedy S.H., Lam R.W., Daskalakis Z.J. (2018). Effectiveness of theta burst versus high-frequency repetitive transcranial magnetic stimulation in patients with depression (THREE-D): A randomised non-inferiority trial. Lancet.

[21] Auvichayapat P., Auvichayapat N. (2009). Basic principle of transcranial magnetic stimulation. J. Med. Assoc. Thai..

[22] Salinas F.S., Lancaster J.L., Fox P.T. (2009). 3D modeling of the total electric field induced by transcranial magnetic stimulation using the boundary element method. Phys. Med. Biol..

[23] Benavides F., Shine M.G., Stefanovic F., Chen R., Jo H.J. (2025). Repetitive transcranial magnetic stimulation for enhancing motor function after spinal cord injury: A narrative review. Front. Neurol..

[24] Wagner T., Valero-Cabre A., Pascual-Leone A. (2007). Noninvasive human brain stimulation. Annu. Rev. Biomed. Eng..

[25] Williams P.I., Marketos P., Crowther L.J., Jiles D.C. (2011). New designs for deep brain transcranial magnetic stimulation. IEEE Trans. Magn..

[26] Rossini P.M., Burke D., Chen R., Cohen L.G., Daskalakis Z., Di Iorio R., Di Lazzaro V., Ferreri F., Fitzgerald P.B., George M.S. (2015). Non-invasive electrical and magnetic stimulation of the brain, spinal cord, roots and peripheral nerves: Basic principles and procedures for routine clinical and research application. An updated report from an IFCN Committee. Clin. Neurophysiol..

[27] Ueno S., Matsuda T., Fujiki M. (1990). Functional mapping of the human motor cortex obtained by focal and vectorial magnetic stimulation of the brain. IEEE Trans. Magn..

[28] Lu M., Ueno S. (2015). Deep transcranial magnetic stimulation using figure-of-eight and halo coils. IEEE Trans. Magn..

[29] O’Reardon J.P., Solvason H.B., Janicak P.G., Sampson S., Isenberg K.E., Nahas Z., McDonald W.M., Avery D., Fitzgerald P.B., Loo C. (2007). Efficacy and safety of transcranial magnetic stimulation in the acute treatment of major depression: A multisite randomized controlled trial. Biol. Psychiatry.

[30] George M.S., Lisanby S.H., Avery D., McDonald W.M., Durkalski V., Pavlicova M., Anderson B., Nahas Z., Bulow P., Zarkowski P. (2010). Daily left prefrontal transcranial magnetic stimulation therapy for major depressive disorder: A sham-controlled randomized trial. Arch. Gen. Psychiatry.

[31] Rossi S., Antal A., Bestmann S., Bikson M., Brewer C., Brockmöller J., Carpenter L.L., Cincotta M., Chen R., Daskalakis J.D. (2021). Safety and recommendations for TMS use in healthy subjects and patient populations, with updates on training, ethical and regulatory issues: Expert Guidelines. Clin. Neurophysiol..

[32] Berlim M.T., Van den Eynde F., Jeff Daskalakis Z. (2013). Clinically meaningful efficacy and acceptability of low-frequency repetitive transcranial magnetic stimulation (rTMS) for treating primary major depression: A meta-analysis of randomized, double-blind and sham-controlled trials. Neuropsychopharmacology.

[33] Fitzgerald P.B., Brown T.L., Marston N.A.U., Daskalakis Z.J., de Castella A., Kulkarni J. (2003). Transcranial magnetic stimulation in the treatment of depression: A double-blind, placebo-controlled trial. Arch. Gen. Psychiatry.

[34] McDonald W.M., Durkalski V., Ball E.R., Holtzheimer P.E., Pavlicova M., Lisanby S.H., Avery D., Anderson B.S., Nahas Z., Zarkowski P. (2011). Improving the antidepressant efficacy of transcranial magnetic stimulation: Maximizing the number of stimulations and treatment location in treatment-resistant depression. Depress. Anxiety.

[35] Berlim M.T., Van den Eynde F., Daskalakis Z.J. (2013). A systematic review and meta-analysis on the efficacy and acceptability of bilateral repetitive transcranial magnetic stimulation (rTMS) for treating major depression. Psychol. Med..

[36] Cole E.J., Stimpson K.H., Bentzley B.S., Gulser M., Cherian K., Tischler C., Nejad R., Pankow H., Choi E., Aaron H. (2020). Stanford accelerated intelligent neuromodulation therapy for treatment-resistant depression. Am. J. Psychiatry.

[37] Luehr J.G., Fritz E., Turner M., Schupp C., Sackeim H.A. (2024). Accelerated transcranial magnetic stimulation: A pilot study of safety and efficacy using a pragmatic protocol. Brain Stimul. Basic Transl. Clin. Res. Neuromodul..

[38] Webster B.R., Celnik P.A., Cohen L.G. (2006). Noninvasive brain stimulation in stroke rehabilitation. NeuroRx.

[39] Nowak D.A., Grefkes C., Ameli M., Fink G.R. (2009). Interhemispheric competition after stroke: Brain stimulation to enhance recovery of function of the affected hand. Neurorehabilit. Neural Repair.

[40] Stewart J., Alabbas K. (2026). Repetitive transcranial magnetic stimulation in post-stroke motor recovery: A narrative review. Cureus.

[41] Chen N., Qiu X., Hua Y., Hu J., Bai Y. (2023). Effects of sequential inhibitory and facilitatory repetitive transcranial magnetic stimulation on neurological and functional recovery of a patient with chronic stroke: A case report and literature review. Front. Neurol..

[42] Luk K.Y., Ouyang H.X., Pang M.Y.C. (2022). Low-frequency rTMS over Contralesional M1 increases Ipsilesional cortical excitability and motor function with decreased interhemispheric asymmetry in subacute stroke: A randomized controlled study. Neural Plast..

[43] Kuzu Ö., Adiguzel E., Kesikburun S., Yaşar E., Yılmaz B. (2021). The effect of sham controlled continuous theta burst stimulation and low frequency repetitive transcranial magnetic stimulation on upper extremity spasticity and functional recovery in chronic ischemic stroke patients. J. Stroke Cerebrovasc. Dis..

[44] Vink J.J.T., van Lieshout E.C.C., Otte W.M., van Eijk R.P., Kouwenhoven M., Neggers S.F., van der Worp H.B., Visser-Meily J.M., Dijkhuizen R.M. (2023). Continuous theta-burst stimulation of the contralesional primary motor cortex for promotion of upper limb recovery after stroke: A randomized controlled trial. Stroke.

[45] Chen Y.J., Huang Y.Z., Chen C.Y., Chen C.-L., Chen H.-C., Wu C.-Y., Lin K.-C., Chang T.-L. (2019). Intermittent theta burst stimulation enhances upper limb motor function in patients with chronic stroke: A pilot randomized controlled trial. BMC Neurol..

[46] Kim W.S., Kwon B.S., Seo H.G., Gil Seo H., Park J., Paik N.-J. (2020). Low-frequency repetitive transcranial magnetic stimulation over contralesional motor cortex for motor recovery in subacute ischemic stroke: A randomized sham-controlled trial. Neurorehabilit. Neural Repair.

[47] Qian W., Liao X., Ju X., Gao Y., Wu M., Xie C., Zhang Y., Long X., Qian S., Gong Y. (2024). Effects of low frequency repetitive transcranial magnetic stimulation on motor recovery in subacute stroke patients with different motor evoked potential status: A randomized controlled trial. Front. Neurol..

[48] Chang W.H., Kim Y.H., Bang O.Y., Kim S., Park Y., Lee P. (2010). Long-term effects of rTMS on motor recovery in patients after subacute stroke. J. Rehabil. Med..

[49] Kim D.H., Shin J.C., Jung S., Jung T.-M., Kim D.Y. (2015). Effects of intermittent theta burst stimulation on spasticity after stroke. Neuroreport.

[50] Noseda R., Burstein R. (2013). Migraine pathophysiology: Anatomy of the trigeminovascular pathway and associated neurological symptoms, cortical spreading depression, sensitization, and modulation of pain. PAIN.

[51] De Tommaso M., Vecchio E., Quitadamo S.G., Coppola G., Di Renzo A., Parisi V., Silvestro M., Russo A., Tedeschi G. (2021). Pain-related brain connectivity changes in migraine: A narrative review and proof of concept about possible novel treatments interference. Brain Sci..

[52] Misra U.K., Kalita J., Bhoi S.K. (2013). High-rate repetitive transcranial magnetic stimulation in migraine prophylaxis: A randomized, placebo-controlled study. J. Neurol..

[53] Shehata H.S., Esmail E.H., Abdelalim A., El-Jaafary S., Elmazny A., Sabbah A., Shalaby N.M. (2016). Repetitive transcranial magnetic stimulation versus botulinum toxin injection in chronic migraine prophylaxis: A pilot randomized trial. J. Pain Res..

[54] Brighina F., Piazza A., Vitello G., Aloisio A., Palermo A., Daniele O., Fierro B. (2004). rTMS of the prefrontal cortex in the treatment of chronic migraine: A pilot study. J. Neurol. Sci..

[55] Conforto A.B., Amaro E., Gonçalves A.L., Mercante J.P., Guendler V.Z., Ferreira J.R., Kirschner C.C., Peres M.F. (2014). Randomized, proof-of-principle clinical trial of active transcranial magnetic stimulation in chronic migraine. Cephalalgia.

[56] Sahu A.K., Sinha V.K., Goyal N. (2019). Effect of adjunctive intermittent theta-burst repetitive transcranial magnetic stimulation as a prophylactic treatment in migraine patients: A double-blind sham-controlled study. Indian J. Psychiatry.

[57] Moisset X., Pereira B., Ciampi de Andrade D., Fontaine D., Lantéri-Minet M., Mawet J. (2020). Neuromodulation techniques for acute and preventive migraine treatment: A systematic review and meta-analysis of randomized controlled trials. J. Headache Pain.

[58] Zhong J., Lan W., Feng Y., Yu L., Xiao R., Shen Y., Zou Z., Hou X. (2022). Efficacy of repetitive transcranial magnetic stimulation on chronic migraine: A meta-analysis. Front. Neurol..

[59] Brys M., Fox M.D., Agarwal S., Biagioni M., Dacpano G., Kumar P., Pirraglia E., Chen R., Wu A., Fernandez H. (2016). Multifocal repetitive TMS for motor and mood symptoms of Parkinson disease: A randomized trial. Neurology.

[60] Song W., Zhang Z., Lv B., Li J., Chen H., Zhang S., Zu J., Dong L., Xu C., Zhou M. (2024). High-frequency rTMS over bilateral primary motor cortex improves freezing of gait and emotion regulation in patients with parkinson’s disease: A randomized controlled trial. Front. Aging Neurosci..

[61] Mi T.M., Garg S., Ba F., Liu A.-P., Wu T., Gao L.-L., Dan X.-J., Chan P., McKeown M.J. (2019). High-frequency rTMS over the supplementary motor area improves freezing of gait in Parkinson’s disease: A randomized controlled trial. Park. Relat. Disord..

[62] Shirota Y., Ohtsu H., Hamada M., Enomoto H., Ugawa Y. (2013). Supplementary motor area stimulation for Parkinson disease: A randomized controlled study. Neurology.

[63] Zhang W., Deng B., Xie F., Zhou H., Guo J.-F., Jiang H., Sim A., Tang B., Wang Q. (2022). Efficacy of repetitive transcranial magnetic stimulation in Parkinson’s disease: A systematic review and meta-analysis of randomised controlled trials. eClinicalMedicine.

[64] Chen Y., Jiang H., Wei Y., Ye S., Jiang J., Mak M.K., Pang M.Y., Gao Q., Huang M. (2025). Effects of non-invasive brain stimulation over the supplementary motor area on motor function in Parkinson’s disease: A systematic review and meta-analysis. Brain Stimul..

[65] Cheng B., Zhu T., Zhao W., Sun L., Shen Y., Xiao W., Zhang S. (2022). Effect of theta burst stimulation-patterned rTMS on motor and nonmotor dysfunction of Parkinson’s disease: A systematic review and metaanalysis. Front. Neurol..

[66] Zhang L., Jiang Y., Fan W., Xue H. (2025). Unveiling the efficacy of repetitive transcranial magnetic stimulation in Parkinson’s disease: A comprehensive review of systematic analyses. PLoS ONE.

[67] Cardoso E.F., Fregni F., Martins Maia F., Boggio P.S., Myczkowski M.L., Coracini K., Vieira A.L., Melo L.M., Sato J.R., Marcolin M.A. (2008). rTMS treatment for depression in Parkinson’s disease increases BOLD responses in the left prefrontal cortex. Int. J. Neuropsychopharmacol..

[68] Nutt D.J., Malizia A.L. (2004). Structural and functional brain changes in posttraumatic stress disorder. J. Clin. Psychiatry.

[69] Watts B.V., Landon B., Groft A., Young-Xu Y. (2012). A sham controlled study of repetitive transcranial magnetic stimulation for posttraumatic stress disorder. Brain Stimul..

[70] Leong K., Chan P., Ong L., Zwicker A., Willan S., Lam R.W., McGirr A. (2020). A Randomized Sham-controlled Trial of 1-Hz and 10-Hz Repetitive Transcranial Magnetic Stimulation (rTMS) of the Right Dorsolateral Prefrontal Cortex in Civilian Post-traumatic Stress Disorder: Un essai randomisé contrôlé simulé de stimulation magnétique transcrânienne repetitive (SMTr) de 1 Hz et 10 Hz du cortex préfrontal dorsolatéral droit dans le trouble de stress post-traumatique chez des civils. Can. J. Psychiatry.

[71] Cohen H., Kaplan Z., Kotler M., Kouperman I., Moisa R., Grisaru N. (2004). Repetitive transcranial magnetic stimulation of the right dorsolateral prefrontal cortex in posttraumatic stress disorder: A double-blind, placebo-controlled study. Am. J. Psychiatry.

[72] Boggio P.S., Rocha M., Oliveira M.O., Fecteau S., Cohen R.B., Campanhã C., Ferreira-Santos E., Meleiro A., Corchs F., Zaghi S. (2010). Noninvasive brain stimulation with high-frequency and low-intensity repetitive transcranial magnetic stimulation treatment for posttraumatic stress disorder. J. Clin. Psychiatry.

[73] Ahmadizadeh M.J., Rezaei M. (2018). Unilateral right and bilateral dorsolateral prefrontal cortex transcranial magnetic stimulation in treatment post-traumatic stress disorder: A randomized controlled study. Brain Res. Bull..

[74] Philip N.S., Barredo J., Aiken E., Larson V., Jones R.N., Shea M.T., Greenberg B.D., Wout-Frank M. (2019). Theta-burst transcranial magnetic stimulation for posttraumatic stress disorder. Am. J. Psychiatry.

[75] Brown R., Cherian K., Jones K., Wickham R., Gomez R., Sahlem G. (2024). Repetitive transcranial magnetic stimulation for post-traumatic stress disorder in adults. Cochrane Database Syst. Rev..

[76] Lontis E.R., Voigt M., Struijk J.J. (2006). Focality assessment in transcranial magnetic stimulation with double and cone coils. J. Clin. Neurophysiol..

[77] MagVenture Cool D-B80[EB/OL]. https://magventure.com/products/cool-d-b80/.

[78] Crowther L.J., Marketos P., Williams P.I., Melikhov Y., Jiles D.C., Starzewski J.H. (2011). Transcranial magnetic stimulation: Improved coil design for deep brain investigation. J. Appl. Phys..

[79] Lu M., Ueno S. (2015). Computational study toward deep transcranial magnetic stimulation using coaxial circular coils. IEEE Trans. Biomed. Eng..

[80] Deng Z.D., Lisanby S.H., Peterchev A.V. (2014). Coil design considerations for deep transcranial magnetic stimulation. Clin. Neurophysiol..

[81] BrainsWay Ltd. TMS Technology: Learn About BrainsWay Deep TMS[EB/OL]. https://www.brainsway.com/how-does-it-work/technology/.

[82] Levkovitz Y., Isserles M., Padberg F., Lisanby S.H., Bystritsky A., Xia G., Tendler A., Daskalakis Z.J., Winston J.L., Dannon P. (2015). Efficacy and safety of deep transcranial magnetic stimulation for major depression: A prospective multicenter randomized controlled trial. World Psychiatry.

[83] Zangen A., Moshe H., Martinez D., Barnea-Ygael N., Vapnik T., Bystritsky A., Duffy W., Toder D., Casuto L., Grosz M.L. (2021). Repetitive transcranial magnetic stimulation for smoking cessation: A pivotal multicenter double-blind randomized controlled trial. World Psychiatry.

[84] Carmi L., Tendler A., Bystritsky A., Hollander E., Blumberger D.M., Daskalakis J., Ward H., Lapidus K., Goodman W., Casuto L. (2019). Efficacy and safety of deep transcranial magnetic stimulation for obsessive-compulsive disorder: A prospective multicenter randomized double-blind placebo-controlled trial. Am. J. Psychiatry.

[85] Harmelech T., Roth Y., Tendler A. (2021). Deep TMS H7 coil: Features, applications & future. Expert Rev. Med. Devices.

[86] Gomez-Tames J., Hamasaka A., Hirata A., Laakso I., Lu M., Ueno S. (2020). Group-level analysis of induced electric field in deep brain regions by different TMS coils. Phys. Med. Biol..

[87] US Food and Drug Administration (2013). 510(k) Summary and Substantial Equivalence Letter K122288: Brainsway Deep TMS System[EB/OL]. https://www.accessdata.fda.gov/scripts/cdrh/cfdocs/cfpmn/pmn.cfm?id=K122288.

[88] Zangen A., Zibman S., Tendler A., Barnea-Ygael N., Alyagon U., Blumberger D.M., Grammer G., Shalev H., Gulevski T., Vapnik T. (2023). Pursuing personalized medicine for depression by targeting the lateral or medial prefrontal cortex with Deep TMS. JCI Insight.

[89] Kaster T.S., Daskalakis Z.J., Noda Y., Knyahnytska Y., Downar J., Rajji T.K., Levkovitz Y., Zangen A., Butters M.A., Mulsant B.H. (2018). Efficacy, tolerability, and cognitive effects of deep transcranial magnetic stimulation for late-life depression: A prospective randomized controlled trial. Neuropsychopharmacology.

[90] Filipčić I., Filipčić I.Š., Milovac Ž., Sučić S., Gajšak T., Ivezić E., Bašić S., Bajić Ž., Heilig M. (2019). Efficacy of repetitive transcranial magnetic stimulation using a figure-8-coil or an H1-Coil in treatment of major depressive disorder; A randomized clinical trial. J. Psychiatr. Res..

[91] Pell G.S., Harmelech T., Zibman S., Roth Y., Tendler A., Zangen A. (2022). Efficacy of deep TMS with the H1 coil for anxious depression. J. Clin. Med..

[92] Lan X.J., Yang X.H., Mo Y., Deng C.-J., Huang X.-B., Cai D.-B., Zheng W. (2024). Deep transcranial magnetic stimulation for treatment-resistant depression: A systematic review and meta-analysis of randomized controlled studies. Asian J. Psychiatry.

[93] US Food and Drug Administration (2022). 510(k) Summary and Substantial Equivalence Letter K220819: BrainsWay Deep TMS™ System[EB/OL]. https://www.accessdata.fda.gov/scripts/cdrh/cfdocs/cfpmn/pmn.cfm?id=K220819.

[94] Carmi L., Alyagon U., Barnea-Ygael N., Zohar J., Dar R., Zangen A. (2018). Clinical and electrophysiological outcomes of deep TMS over the medial prefrontal and anterior cingulate cortices in OCD patients. Brain Stimul..

[95] US Food and Drug Administration (2017). De Novo Summary DEN170078: Brainsway Deep Transcranial Magnetic Stimulation System[EB/OL]. https://www.accessdata.fda.gov/scripts/cdrh/cfdocs/cfPMN/denovo.cfm?id=DEN170078.

[96] Roth Y., Tendler A., Arikan M.K., Vidrine R., Kent D., Muir O., MacMillan C., Casuto L., Grammer G., Sauve W. (2021). Real-world efficacy of deep TMS for obsessive-compulsive disorder: Post-marketing data collected from twenty-two clinical sites. J. Psychiatr. Res..

[97] Li K., Qian L., Zhang C., Li R., Zeng J., Xue C., Deng W. (2024). Deep transcranial magnetic stimulation for treatment-resistant obsessive-compulsive disorder: A meta-analysis of randomized-controlled trials. J. Psychiatr. Res..

[98] Grassi G., Moradei C., Cecchelli C. (2023). Will transcranial magnetic stimulation improve the treatment of obsessive–compulsive disorder? A systematic review and meta-analysis of current targets and clinical evidence. Life.

[99] Kar S.K., Agrawal A., Silva-dos-Santos A., Gupta Y., Deng Z.-D. (2024). The efficacy of transcranial magnetic stimulation in the treatment of obsessive-compulsive disorder: An umbrella review of meta-analyses. CNS Spectr..

[100] (2020). NICE Transcranial Magnetic Stimulation for Obsessive-Compulsive Disorder: HTG548[R/OL]. https://www.nice.org.uk/guidance/htg548.

[101] Fiocchi S., Chiaramello E., Luzi L., Ferrulli A., Bonato M., Roth Y., Zangen A., Ravazzani P., Parazzini M. (2018). Deep transcranial magnetic stimulation for the addiction treatment: Electric field distribution modeling. IEEE J. Electromagn. Microw. Med. Biol..

[102] Dinur-Klein L., Dannon P., Hadar A., Rosenberg O., Roth Y., Kotler M., Zangen A. (2014). Smoking cessation induced by deep repetitive transcranial magnetic stimulation of the prefrontal and insular cortices: A prospective, randomized controlled trial. Biol. Psychiatry.

[103] US Food and Drug Administration (2020). 510(k) Substantial Equivalence Determination Letter K200957: Brainsway Deep TMS System[EB/OL]. https://www.accessdata.fda.gov/scripts/cdrh/cfdocs/cfpmn/pmn.cfm?id=K200957.

[104] Bellini B.B., Scholz J.R., Abe T.O., Arnaut D., Tonstad S., Alberto R.L., Gaya P.V., de Moraes I.R., Teixeira M.J., Marcolin M.A. (2024). Does deep TMS really works for smoking cessation? A prospective, double blind, randomized, sham controlled study. Prog. Neuro-Psychopharmacol. Biol. Psychiatry.

[105] Ibrahim C., Tang V.M., Blumberger D.M., Malik S., Tyndale R.F., Trevizol A.P., Barr M.S., Daskalakis Z.J., Zangen A., Le Foll B. (2023). Efficacy of insula deep repetitive transcranial magnetic stimulation combined with varenicline for smoking cessation: A randomized, double-blind, sham controlled trial. Brain Stimul..

[106] Harel M., Perini I., Kämpe R., Alyagon U., Shalev H., Besser I., Sommer W.H., Heilig M., Zangen A. (2022). Repetitive transcranial magnetic stimulation in alcohol dependence: A randomized, double-blind, sham-controlled proof-of-concept trial targeting the medial prefrontal and anterior cingulate cortices. Biol. Psychiatry.

[107] Sanna A., Fattore L., Badas P., Corona G., Cocco V., Diana M. (2019). Intermittent theta burst stimulation of the prefrontal cortex in cocaine use disorder: A pilot study. Front. Neurosci..

[108] Mehta D.D., Praecht A., Ward H.B., Sanches M., Sorkhou M., Tang V.M., Steele V.R., Hanlon C.A., George T.P. (2024). A systematic review and meta-analysis of neuromodulation therapies for substance use disorders. Neuropsychopharmacology.

[109] Isserles M., Shalev A.Y., Roth Y., Peri T., Kutz I., Zlotnick E., Zangen A. (2013). Effectiveness of deep transcranial magnetic stimulation combined with a brief exposure procedure in post-traumatic stress disorder—A pilot study. Brain Stimul..

[110] Isserles M., Tendler A., Roth Y., Bystritsky A., Blumberger D.M., Ward H., Feifel D., Viner L., Duffy W., Zohar J. (2021). Deep transcranial magnetic stimulation combined with brief exposure for posttraumatic stress disorder: A prospective multisite randomized trial. Biol. Psychiatry.

[111] Lu M., Ueno S., Thorlin T., Persson M. (2009). Calculating the current density and electric field in human head by multichannel transcranial magnetic stimulation. IEEE Trans. Magn..

[112] Demchenko I., Al-Shamali H.F., Rueda A., Tailor I., Janssen-Aguilar R., Schweizer T.A., Dunlop K., Graham S.J., Bhat V. (2025). Magnetic Resonance Imaging-Guided Neuronavigational for Transcranial Magnetic Stimulation in Mood Disorders: Technical Foundation, Advances, and Emerging Tools. Hum. Brain Mapp..

